# The haplotype-resolved genome of *Swinglea glutinosa* and pangenome analysis provide insight into resistance to citrus Huanglongbing

**DOI:** 10.3389/fpls.2026.1988077

**Published:** 2026-10-14

**Authors:** Jiawen Wen, Jin Xu, Connor G. Hendrich, Zhiqian Pang, Nian Wang

**Affiliations:** 1 Citrus Research and Education Center, Institute of Food and Agricultural Sciences, University of Florida, Lake Alfred, FL, United States; 2 Department of Plant Pathology, Institute of Food and Agricultural Sciences, University of Florida, Lake Alfred, FL, United States; 3 Department of Microbiology and Cell Science, Institute of Food and Agricultural Sciences, University of Florida, Lake Alfred, FL, United States

**Keywords:** citrus, HLB, Liberibacter, resistance, susceptibility

## Abstract

Huanglongbing (HLB), caused by *Candidatus* Liberibacter asiaticus (*C*Las), is the most devastating citrus disease worldwide, and all commercial varieties are susceptible to HLB. *Swinglea glutinosa*, a citrus relative, is resistant to *C*Las, but the genetic basis of its HLB resistance remains unknown. We completed a high-quality, haplotype-phased chromosome-level genome of *S. glutinosa.* We conducted a pangenome analysis with 25 citrus and citrus-relative accessions and identified a pan-NLRome of nucleotide-binding domain leucine-rich repeat (NLR) immune receptors representing 19,476 NLR genes. Genes specific to HLB-resistant citrus genotypes were enriched for CoA-ligase activity and auxin transport, and HLB-resistant genotypes consistently carried fewer NLRs. *S. glutinosa* additionally showed significant expansion of *Pathogenesis-Related Protein 1* (*PR1*) genes, lineage-specific antimicrobial peptides, and unique genes enriched for the cullin-RING ubiquitin ligase complex. In susceptible *Citrus sinensis* most differential NLR expression occurred at late infection stages, predominantly among coiled-coil NLRs. A single core CNL orthogroup, expanded in *C. sinensis* and uniformly induced upon infection, accounted for most of this response, indicating that NLR expansion and induction are not sufficient for resistance to HLB. These results will support future functional studies of HLB resistance mechanisms and provide genomic information to facilitate development of HLB-resistant citrus varieties through traditional and biotechnological approaches.

## Introduction

Citrus Huanglongbing (HLB), also known as citrus greening disease, is the most devastating citrus disease and causes tremendous economic losses worldwide (Bové, 2006; Wang et al., 2024b). HLB is caused by the phloem-colonizing bacterial pathogens *Candidatus* Liberibacter asiaticus (*C*Las), *Ca*. L. americanus (*C*Lam), and *Ca*. L. africanus (*C*Laf). *C*Las is the most prevalent HLB pathogen globally and is transmitted by the Asian citrus psyllid (ACP), *Diaphorina citri* (Bové, 2006). ACP also transmits *C*Lam. *C*Las is present in Asia, North America, South America, and Africa in over 60 citrus producing countries and regions including the top citrus producers China, Brazil, United States, India, and Mexico. Most commercial citrus varieties are susceptible to HLB, showing symptoms including blotchy mottling and chlorosis of leaves, twig dieback, root loss, and small, irregularly shaped fruits. Currently, there are no efficient HLB control approaches once citrus trees become infected by *C*Las (Li et al., 2019). Comprehensive region-wide application of psyllid control, removal of diseased trees and replantation has been able to maintain a profitable production in Brazil and China (Yuan et al., 2021) despite the high cost. However, HLB outbreaks were reported in multiple provinces in China including Jiangxi and Guangxi in 2024 and HLB incidence has reached approximately 44.35% in citrus belt of Brazil (Pérez-Hedo et al., 2025). An HLB incidence of 30% has been suggested to be the threshold for groves becoming non-profitable in China (Yuan et al., 2021). In addition, the insect vectors of *C*Las and *C*Laf have been expanding to citrus production areas where they were not present before. For instance, the *C*Laf vector *Trioza erytreae* has spread to Portugal and Spain (Ruíz-Rivero et al., 2021) and *D. citri* has been established in Israel and Cyprus (Serra et al., 2026), posing a tremendous threat to citrus production in the Mediterranean region.

Disease-resistant crops are the most economical and effective solution for disease control. Even though none of the commercial citrus varieties are resistant to HLB, some citrus species and relatives such as Australian desert lime (*C. glauca*), Australian finger lime (*C. australasica*), Australian round lime (*C. australis*), Australian large-leaf wild lime (*C. inodora*), Orange jasmine (*Murraya paniculata*) and *Swinglea glutinosa* were reported to be resistant to HLB (**Table 1**) (Alves et al., 2020; Folimonova et al., 2009; Ramadugu et al., 2016b). These wild citrus species and their relatives are being used to breed HLB-resistant citrus varieties through the traditional breeding approach. Significant effort has been made to understand the resistance mechanism of these citrus relatives, which is critical for breeding HLB-resistant citrus varieties using both traditional and biotechnological approaches (Huang et al., 2021, Huang et al., 2023a, Liu J. et al., 2025). The genome sequences of six HLB-resistant Australian wild limes including *C. australasica*, *C. australis*, *C. garrawayi*, *C. glauca*, and *C. inodora* were decoded (Nakandala et al., 2024a, 2024b, 2023; Singh et al., 2024), providing useful insights regarding the resistance mechanism against HLB. Many species-specific genes for antimicrobial peptides, defense, and volatile compounds were identified in *C. australis* and *C. australasica* (Nakandala et al., 2024b, 2023). A recent study identified a novel class of stable antimicrobial peptides (MaSAMPs) containing α-helix-2 domain from *C. australasica* that might contribute to its resistance against *C*Las by inhibiting its growth and activating host immunity to prevent new infections (Huang, C. Y. et al., 2021). Its homologs have been identified in another HLB-resistant citrus genotype *C. australis* (Nakandala et al., 2023).

**Table 1:** The summary of the 25 citrus and citrus relative accessions.

Species	Name	Common name	HLB resistance	Reference
*Atalantia buxifolia*	ABU	Chinese box orange	Tolerant (T)	Folimonova et al., 2009
*C. glauca*	ADL	Australian desert lime	Resistant (R)	Alves et al., 2020; Ramadugu et al., 2016a
*C. australasica*	AFL	Australian finger lime	R	Alves et al., 2020; Ramadugu et al., 2016a
*C. australis*	ARL	Australian round lime	R	Alves et al., 2020; Ramadugu et al., 2016a
*C. inodora*	AWL	Australian large-leaf wild lime	R	Alves et al., 2020; Ramadugu et al., 2016a
*C. sinensis* ‘Jinhong Bingtang’	BTC	Sweet orange	Susceptible (S)	Folimonova et al., 2009
*C. reticulata* cv. Chachiensis	Cch	mandarin orange	S	Folimonova et al., 2009
*C. clementina*	CCLE	nules clementine	S	Folimonova et al., 2009
*C. honghenensis*	CHH	Honghecheng	NA	NA
*C. hindsii*	CHIN	Kumquat	S	Folimonova et al., 2009
*C. ichangensis*	CIC	papeda	T	Wang et al., 2017
*C. limon*	CLM	lime	S	Ramadugu et al., 2016a
*C. medica*	CITME	citron	S	Ramadugu et al., 2016a
*C. maxima* ‘Cupi Majiayou’	CMJ	pummelo	S	Ramadugu et al., 2016a
*C. reticulata* (wild mandarin)	CRE	mandarin	S	Ramadugu et al., 2016a
*C. sinensis* (Di-Haploid Sweet Orange)	Cs	Sweet orange	S	Folimonova et al., 2009, Ramadugu et al., 2016a
*C. sinensis* cv. Valencia	DVS	Sweet orange	S	Folimonova et al., 2009; Ramadugu et al., 2016a
*C. trifoliata*	CTR	trifoliate	T	Folimonova et al., 2009; Ramadugu et al., 2016a
*C. maxima ‘* *Wanbaiyou* *’*	HWB	pummelo	S	Ramadugu et al., 2016a
*C. maxima* ‘Huazhouyou-tomentosa’	HZY	pummelo	S	Ramadugu et al., 2016a
*C. linwuensis*	LW		NA	NA
*M. paniculata*	MPA	Orange jasmine	R	Ramadugu et al., 2016a; Cifuentes-Arenas et al., 2019
*C. mangshanensis*	MSYG		NA	NA
*S. glutinosa*	Sg		R	Cifuentes-Arenas et al., 2019
*C. maxima* ‘Xipi Majia’	XGF	pummelo	S	Ramadugu et al., 2016a

*S. glutinosa* is the only species in the Rutaceae genus *Swinglea* and was reported to originate from the Philippines. *S. glutinosa* has unique fruit structures, and its floral anatomy is similar to primitive citrus fruit tree. *S. glutinosa* can also be used as a rootstock for other citrus species (Cifuentes-Arenas et al., 2019), suggesting a relatively close relationship between citrus and *S. glutinosa.* Importantly, attempts to inoculate *S. glutinosa* with *C*Las using infected psyllids or grafting only produced rare and transient infections (Cifuentes-Arenas et al., 2019), suggesting that *S. glutinosa* is resistant to *C*Las. However, the resistance mechanism in *S. glutinosa* against *C*Las remains unknown.

In this study, we first sequenced the genome of *S. glutinosa*. By integrating PacBio HiFi and Hi-C sequencing, we generated a high-quality, haplotype-phased, chromosome-level genome assembly of *S. glutinosa*. We then conducted comparative genomic analyses with HLB-resistant and susceptible citrus genotypes to investigate potential genetic mechanisms underlying HLB resistance.

## Results

### Phased chromosome-level genome assembly of *S. glutinosa*

We isolated and sequenced genomic DNA from *S. glutinosa* leaf tissue. We obtained 27 Gb of HiFi long-reads with a read N50 length of 17 kb, resulting in 88-fold coverage of the *S. glutinosa* genome. A collapsed haploid genome was assembled firstly using the HiFi data. The assembly has a total size of 284.6 Mb with a contig N50 of 22 Mb (**Table 2**). BUSCO analysis revealed that 98.5% of conserved single-copy orthologs were present in the assembly, indicating a high level of gene-space completeness. The short-read mapping rate was used to evaluate the assembly accuracy. Approximately 99.63% of short reads could be mapped to the collapsed assembly.

**Table 2:** The summary of the *S. glutinosa* genome.

Genome assembly	collapsed	hapA (1)	hapB (2)
Size (Mb)	284.6	300.38	287.09
Contig N50 (Mb)	22	17.24	20
Longest contig (Mb)	48.09	47.56	47.67
Complete BUSCOs	98.50%	98.50%	98.70%
GC content	33.9	34.27	34.03
Contig N50 (Mb, after Hi-C)	30.84	27.99	30.71
Longest scaffold (Mb, after Hi-C)	48.10	47.56	47.67
Genome annotation			
LTR	28.38%	33.75%	34.91%
DNA elements	5.57%	5.05%	4.33%
LINE	1.80%	1.09%	0.93%
MITE	0.05%	0.06%	0.06%
Helitron	0.97%	0.17%	0.20%
SINE	0.00%	0.02%	0.04%
Unclassified	14.86%	10.94%	10.77%
Satellite	0.02%	0.70%	0.38%
Simple repeat	0.01%	0.01%	0.01%
Number of protein-coding genes	27,129	30,004	27,798
Complete BUSCOs	96.20%	96.00%	95.90%
Average length of mRNA (bp)	2607.47	2414.57	2538.06
Average length of CDS (bp)	1128.42	1101.99	1028.33
Mean exon length (bp)	220.04	232.49	221.13
Average of exon number	5.13	4.74	3.87

A Hi-C integrated method was used to assemble two haplotype assemblies (**Figure 1A** and **Supplementary Figure S1**; **Table 2**). Due to many telomeric or knob repeats in the plant genome, we only used a total of 46 × coverage of the HiFi data, which prevented producing many fragment contigs in the assemblies. A total of 169.8 Gb Hi-C data was used to assemble the high-quality haplotype assemblies. Consequently, the size of haplotype A is 300.38 Mb with a contig N50 of 17.24 Mb and the haplotype B is 287.09 Mb in length with a contig N50 of 20 Mb. The k-mer spectrum analysis revealed that the two haplotypes are well balanced (**Supplementary Figure S1**). BUSCO analysis showed that both haplotypes reached more than 98% completeness. A total of 99.64% short reads could be aligned to the two assemblies.

**Figure 1:**
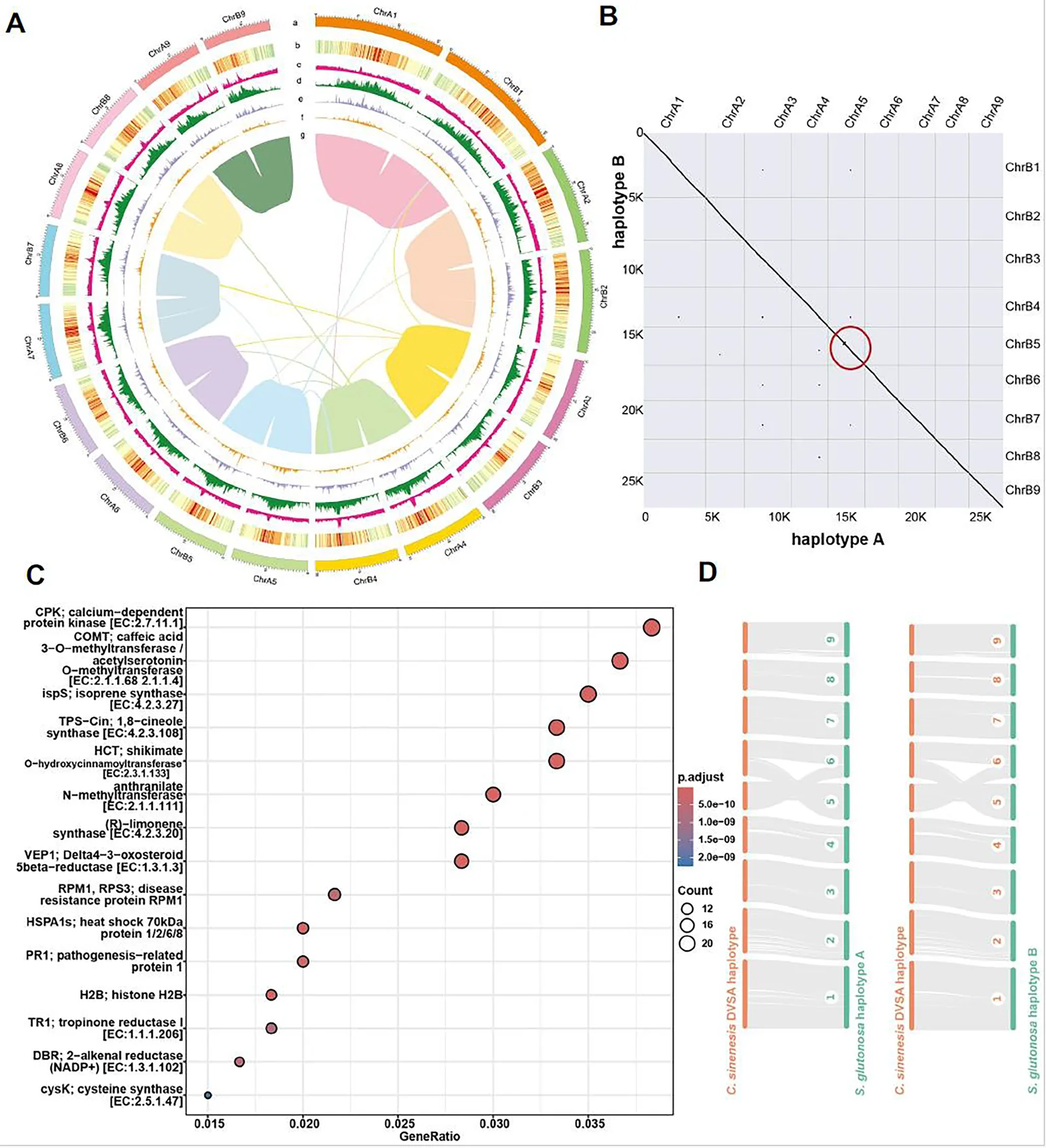
Genome landscape of *S. glutinosa* and comparative genomic analysis of *S. glutinosa* and other citrus species. **(A)** Genomic features of *S. glutinosa*. a-g pseudochromosomes, gene density, GC content, LTR density, Copia density, Gypsy density, genome synteny, respectively. **(B)** Genome synteny analysis between haplotype A (1) and haplotype B (2). The large chromosomal inversion indicated is circled. **(C)** KEGG enrichment analysis of gene families expanded in *S. glutinosa*. **(D)** Genome synteny analysis between *C. sinensis* DVS haplotype A and the two haplotype assemblies of *S. glutinosa*.

To obtain the chromosome-level assemblies, a total of 70 Gb Hi-C data was used to scaffold these three assemblies separately. Consequently, the scaffold N50 values of the final collapsed, haplotype A and B genome assemblies were 30.84 Mb, 27.99 Mb and 30.71 Mb, respectively. In the collapsed genome, 99.9% of the sequences could be anchored into 9 pseudochromosomes. In the assemblies of haplotype A and B, 93.17% and 97.87% sequences could be successfully anchored (**Supplementary Figure S2**). We also observed that the two haplotypes showed high level of collinearity except a large inversion occurred at chromosome 5 from 7.5 Mb to 9 Mb (**Figure 1B**; **Supplementary Figure S3**).

### Genome annotation

More than 50% of the *S. glutinosa* genome are repeat sequences. Long terminal repeat (LTR) retrotransposons are the predominant group with 28.38%, 33.75% and 34.91% in the collapsed, haplotype A and haplotype B assemblies, respectively (**Table 2**). The DNA elements and the unclassified repeats cover 4.33%–5.57% and 10.77%–14.86% of the genome, respectively.

A total of 27,129, 30,004, and 27,798 protein-coding genes were predicted in the collapsed, haplotype A and haplotype B assemblies, respectively. In the collapsed genome, the average lengths of the predicted gene models, CDSs, and exons were 2607 bp, 1128 bp, and 220 bp, respectively. BUSCO analysis revealed that all the assemblies reached more than 95% completeness, indicating high quality of the assemblies. For functional annotation, more than 90% of the genes could be assigned to KEGG terms and GO terms. A total of 1826 transcription factors were identified, with AP2/ERF-ERF (110) accounting for the largest group.

### Comparative genomics analysis

Whole genome duplication (WGD) analysis showed that *S. glutinosa* contains the shared ancient WGD that occurred within eudicots (**Supplementary Figure S4**) (Wu et al., 2020). *S. glutinosa* consists of 188 and 350 significantly expanded and contracted gene families, respectively (*P* < 0.01) (**Supplementary Tables S1 and S2**). According to the functional enrichment results, the expanded gene families were enriched in KO terms including “disease resistance protein RPM1”, “heat shock 70 kDa protein” and “pathogenesis-related protein 1 (PR1)” (**Figure 1C**; **Supplementary Table S3**), as well as GO terms including “calmodulin-dependent protein kinase activity”, “calcium-dependent protein serine/threonine kinase activity” and “calcium-dependent protein kinase activity” (**Supplementary Figure S5**).

According to the genome synteny analysis, the chromosomes of *S. glutinosa* also showed high collinearity with *C. sinensis* cv. Valencia (DVS) except the large structural variations on the chromosomes 5 and 6 (**Figure 1D**).

### Pangenome analysis

A pangenome analysis of the orange subfamily was previously conducted using 18 genomes (Huang et al., 2023b). Here we expanded this analysis to genome assemblies from 25 citrus and citrus-relative accessions, comprising the newly sequenced *S. glutinosa* and 24 HLB-resistant, tolerant and susceptible genotypes with complete genomes (**Table 1**). Haplotype-resolved genomes contributed one assembly per haplotype. The pangenome reached a plateau (**Figure 2A**), indicating that the gene family space of citrus was completely covered. Of the 40,756 gene families identified, 23.7% were present in all assemblies (core) and 5.8% in more than 90% (softcore); 64.1% were present in 2 to 28 assemblies (dispensable) and 6.5% in a single assembly (species- or haplotype-specific). Among the individual genotypes, *C. australasica* carried the largest number of private genes, followed by *C. glauca* (**Figure 2B**).

**Figure 2:**
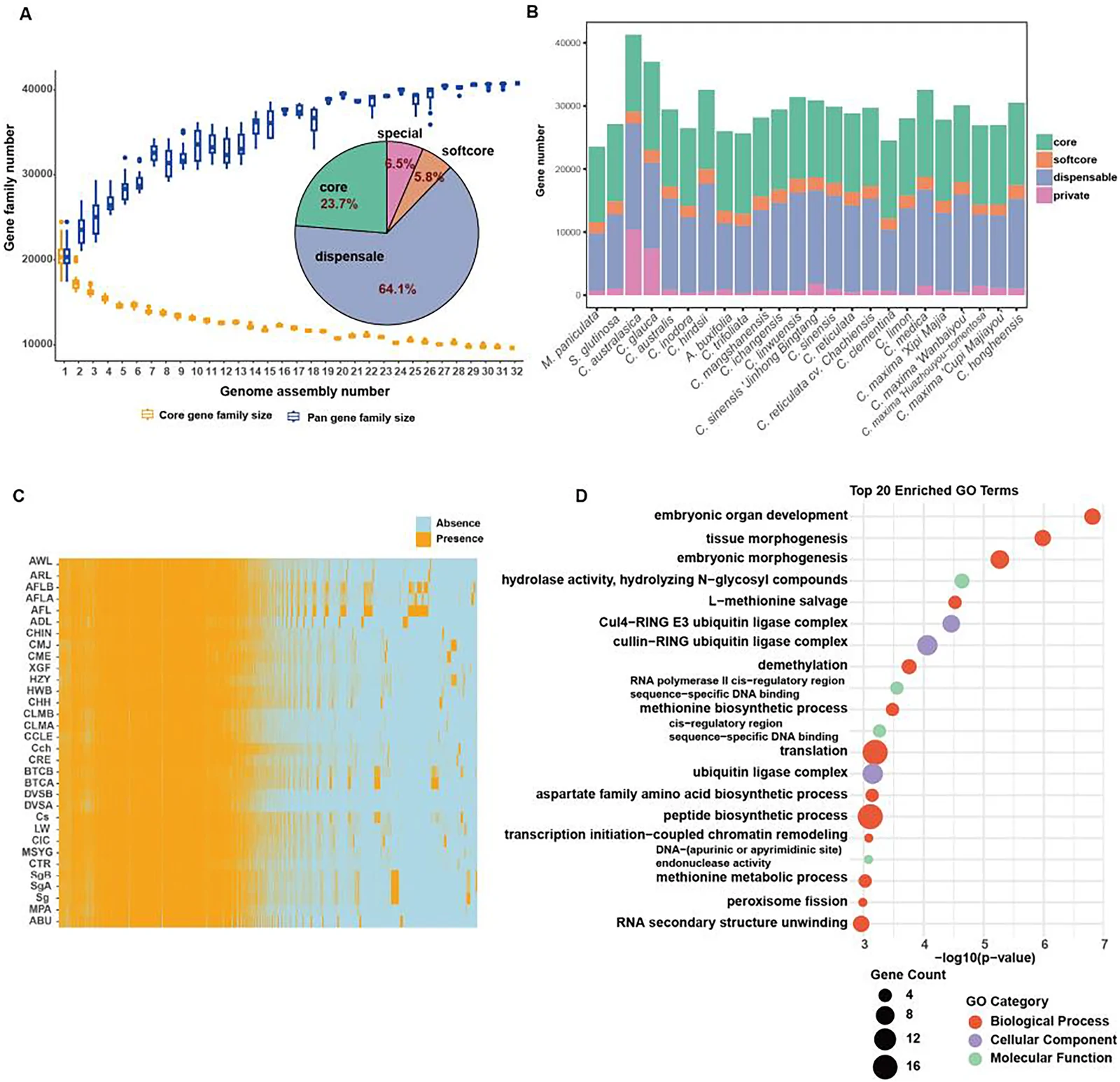
Pangenome analysis of *S. glutinosa* and other citrus species and relatives. **(A)** Influence of the number of genome assemblies on the core gene family size and the pan gene family size. **(B)** The gene composition of the 24 citrus species and relatives. *C. sinensis* (DVS) was not included. **(C)** Gene family presence-absence analysis on *S. glutinosa* and other accessions. **(D)** GO enrichment analysis of unique gene families in *S. glutinosa*.

Gene presence–absence analysis placed the gene-family composition of *S. glutinosa* closer to that of *M. paniculata* than to any other species, and the collapsed assembly and the two haplotype assemblies of *S. glutinosa* were highly concordant (**Figure 2C**). The *S. glutinosa* collapsed assembly carried 1,089 unique genes. Among biological processes, peptide biosynthetic process and translation were enriched, among molecular functions, hydrolase activity and cis-regulatory region sequence-specific DNA binding were enriched, whereas among cellular components, the enriched included the Cul4-RING E3 ubiquitin ligase complex, the cullin-RING ubiquitin ligase complex and the ubiquitin ligase complex (**Figure 2D**; **Supplementary Table S4**).

To identify genes associated with HLB resistance, we compared genotypes in two groups defined by HLB responses reported in the literature: resistant (*C. australasica*, *C. glauca*, *C. australis*, *C. inodora*, *S. glutinosa* and *M. paniculata*) and susceptible (**Table 1**). Tolerant genotypes such as *C. trifoliata* and *A. buxifolia* were excluded to simplify the analysis. A total of 2,017 gene families were specific to the resistant group (**Supplementary Table S5**). The strongest enrichments were observed for CoA-ligase activity, most pronounced in the Australian limes, and for auxin transport, including auxin transmembrane transporter activity and auxin export across the plasma membrane. Enrichments were also observed for oxidative stress response (glyoxylate reductase (NADPH) activity), glucose metabolism (intracellular glucose homeostasis, cellular response to glucose stimulus), photorespiration (hydroxypyruvate reductase and glycerate dehydrogenase activity) and gibberellic acid signaling (**Supplementary Table S6**). Lineage-specific signals were also observed: the Australian lime genotypes were enriched for very-long-chain fatty acid-CoA ligase activity, manganese ion binding, auxin response factor pathways and LysM domain receptor-like kinases, whereas *S. glutinosa* was enriched for the Cul4A-RING E3 ubiquitin ligase complex and amino acid transaminase activity. A substantial proportion of resistant-group-specific gene families could not be functionally annotated (**Supplementary Table S6**).

### Genome-guided annotation substantially expands the citrus NLR repertoire

Nucleotide-binding leucine-rich repeat (NLR) proteins detect pathogens and activate immune responses in plants. NLR identification based on standard genome annotations gave inconsistent counts within single genotypes. These included for example, annotations across *C. sinensis* DVS haplotype assemblies, suggesting that NLRs are underrepresented in conventional gene models, possibly because the tandem-repeat structure of LRR domains resembles transposable elements and is masked during repeat annotation. We therefore applied an NLR-specific *de novo*, genome-guided annotation to 29 citrus and citrus-relative accessions, recovering 24,719 NLRs (**Supplementary Table S7**), a 2- to 15-fold increase over protein-based prediction (**Supplementary Table S8**). Counts stabilized within genotypes across haplotype assemblies: NLRs in *C. sinensis* ranged from 643 to 672 across four haplotype pairs. *C. maxima* genotypes showed the lowest counts under protein-based prediction and were predicted to carry 706–910 NLRs.

Newly identified NLRs were predominantly from intergenic regions of the standard gene models, averaging 54% across accessions and reaching 85% in individual genomes. Overlap with repeat elements was low (0–5.9% per assembly), and 0.9–32.8% overlapped with non-NLR gene models. In *S. glutinosa*, haplotype assemblies SgA and SgB carried 523 and 525 NLRs, of which 366 and 334 were newly identified from intergenic regions.

Underrepresentation of NLRs in standard annotations therefore reflects the training quality of the gene-model datasets used for protein-coding prediction rather than repeat masking, and NLR-specific annotation is required for accurate NLR cataloguing.

### Citrus Pan-NLRome

For comparative analysis we retained the collapsed assembly, or haplotype A/haplotype 1 of phased assemblies, for each of the 29 accessions. NLRs from the two identification rounds were merged and redundant predictions collapsed by retaining the longest model, generating apan-NLRome of 19,476 NLRs in 898 orthogroups. Of these orthogroups, 76 (8.5%) were core and 71 (7.9%) were softcore, while 398 (44.3%) were dispensable and 353 (39.3%) were private.

NLRs were classified into eight groups: TIR-NLR (TNL), CC-NLR (CNL), CC-RPW8-NLR (RNL), solo NB-ARC (N), TIR–NB-ARC (TN), CC–NB-ARC (CN), RPW8-like CC–NB-ARC (RN), and NB-ARC–LRR (NL) (**Figure 3**). CNLs were the largest group across citrus. HLB-resistant and tolerant species carried consistently fewer NLRs than susceptible species, with resistant genotypes having 319–525 per genome (with an average of 427) and susceptible species harboring 478–910 per genome (with an average of 679). The same contrast was observed for TIR-type receptors: with resistant species carrying 56–88 per genome (average of 71) and susceptible carrying 63–171 per genome (average of 110).

**Figure 3:**
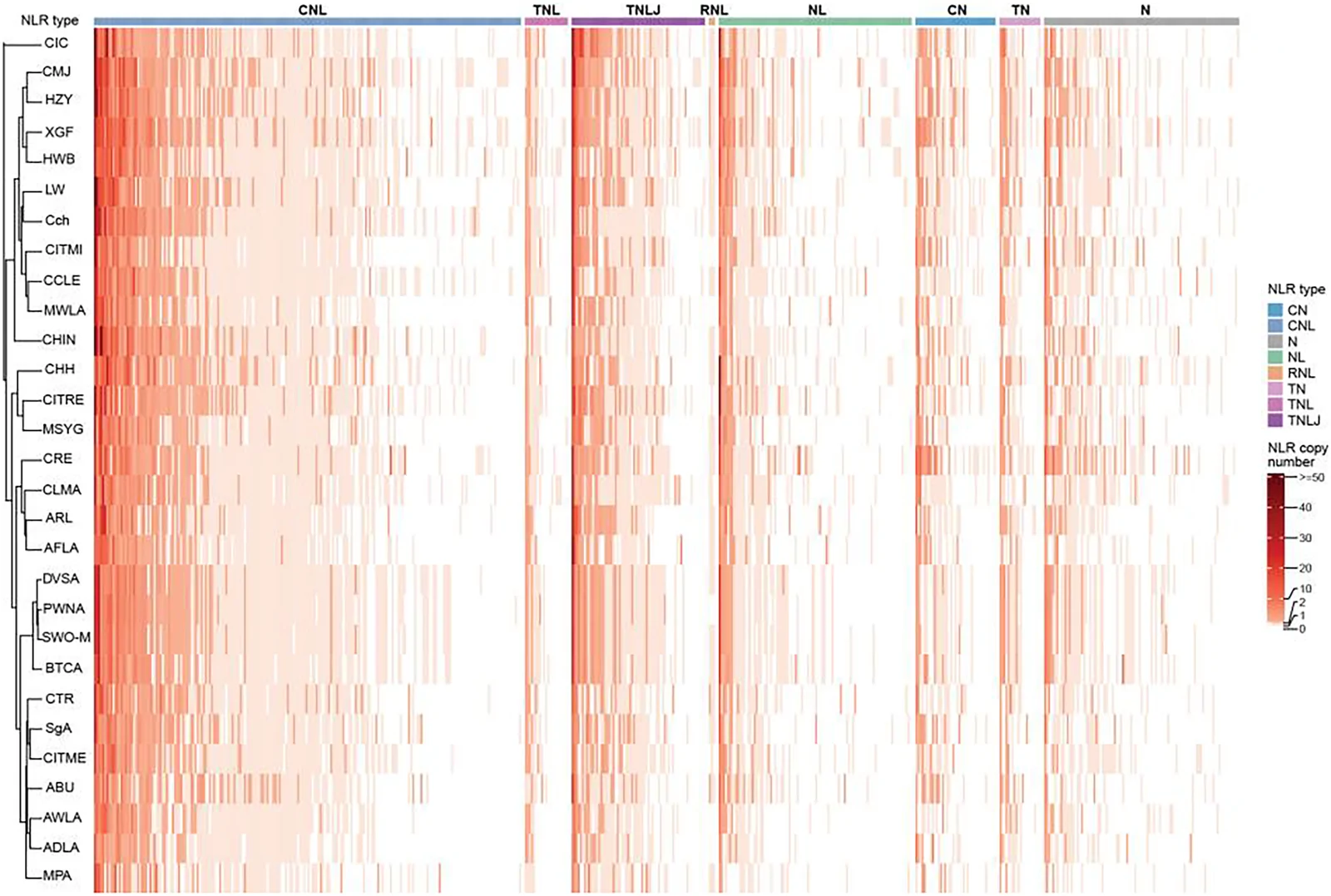
The heatmap of citrus pan-NLRome with 29 accessions included. TNL, TIR-NLR (TNL); CNL, CC-NLR; RNL, CC-RPW8-NLR; N, solo NB-ARC domain; TN, TIR domain combined with NB-ARC domain, TNLJ, TNL with CJID domain and NL, NB-ARC domain combined with leucine-rich repeat. CIC, *C. ichangensis*; CMJ, *C. maxima* ‘Cupi Majiayou’; HZY, *C. maxima* ‘Huazhouyou-tomentosa’; XGF, *C. maxima* ‘Xipi Majia’; HWB, *C. maxima* ‘Wanbaiyou’; LW, *C. linwuensis*; Cch, *C. reticulata* cv. Chachiensis; CITMI, *C. micrantha*; CCLE, *C. clementina*; MWLA, *C. garrawayi* haplotype A; CHIN, *C. hindsii*; CHH, *C. hongheensis*; CITRE, *C. reticulata* (Cleopatra mandarin); MSYG, *C. mangshanensis*; CRE, *C. reticulata* (wild mandarin); CLMA, *C.limon* haplotype A; ARL, *C. australis*; AFLA, *C. australasica* haplotype A; DVSA, *C. sinensis* cv. Valencia haplotype A; PWNA, *C. sinensis* ‘Parent Washington navel’ haplotype A; SWO-M, *C. sinensis* (mandarin haplotype); BTCA, *C. sinensis* ‘Jinhong Bingtang’ haplotype A; CTR, *C. trifoliata*; SgA, *S. glutinosa* haplotype A; CITME, *C. medica*; ABU, *A. buxifolia*; AWLA, *C. inodora* haplotype A; ADLA, *C. glauca* haplotype A; MPA, *M. paniculata*.

Tolerant *C. ichangensis* encodes 54 TNLs and 150 TNLJs (TNL with a CJID motif) and susceptible *C. maxima* ‘Cupi Majiayou’ contains 45 TNLs and 125 TNLJs, versus 13–16 TNLs and 40–48 TNLJs in resistant *C. glauca*. Susceptible *C. sinensis* carried 23–25 TNL and 91–105 TNLJ (**Supplementary Table S7**).

Hierarchical clustering of accessions by NLR orthogroup composition recovered species-level structure: *C. sinensis* varieties grouped together, as did *C. maxima* accessions (**Figure 3**). Resistance status, however, did not structure the tree. *C. australasica* (AFLA) and *C. australis* (ARL) fell in a clade with *C. limon* (CLMA) and wild mandarin *C. reticulata* (CRE), while *C. medica* (CITME) resembled resistant and tolerant species including *S. glutinosa* (SgA) and *C. trifoliata* (CTR).

Several CNL orthogroups were expanded in *C. sinensis*, *C. reticulata* and *C. maxima* relative to citrus relatives. TNL expansion was likewise found in most susceptible species, but also in *C. australasica* and *C. australis*; the core TNLJ orthogroup carrying the CJID domain (OG0000001) was significantly expanded in *C. ichangensis*, *C. australasica*, *C. australis*, *C. sinensis*, *C. reticulata* and *C. maxima* (**Supplementary Figure S6**).

### Expression patterns of NLRs

NLR transcripts are generally assumed to accumulate at low levels in the absence of pathogen challenge (van Wersch et al., 2020). To examine this in citrus, we analyzed the expression of NLRs belonging to pangenome core gene families across a published *C. reticulata* tissue panel of 200 RNA-seq libraries spanning leaves, flowers, fruit, peel, pulp and seeds (Wen et al., 2024). A substantial fraction of NLRs were expressed at moderate to high levels, with the highest levels in young fruit and mature leaves, followed by peel and pulp, and the lowest in seeds with few exceptions (**Supplementary Figure S7**; **Supplementary Table S9**). Across leaf developmental stages, NLR expression was lower in tender leaves, resembling the pattern in flowers.

To examine how citrus NLRs respond to infection, we analyzed published RNA-seq data from *C*Las-infected and healthy *C. sinensis* leaves sampled at 8, 18, 26 and 46 weeks post-graft-inoculation with *C*Las (wpg) (Chin et al., 2020). Differentially expressed NLRs were observed over the course of infection: 8 at 8 wpg, none at 18 wpg, 33 at 26 wpg and 87 at 46 wpg (**Figure 4**), predominantly upregulated at the two late time points. CNLs dominated the response at every stage.

**Figure 4:**
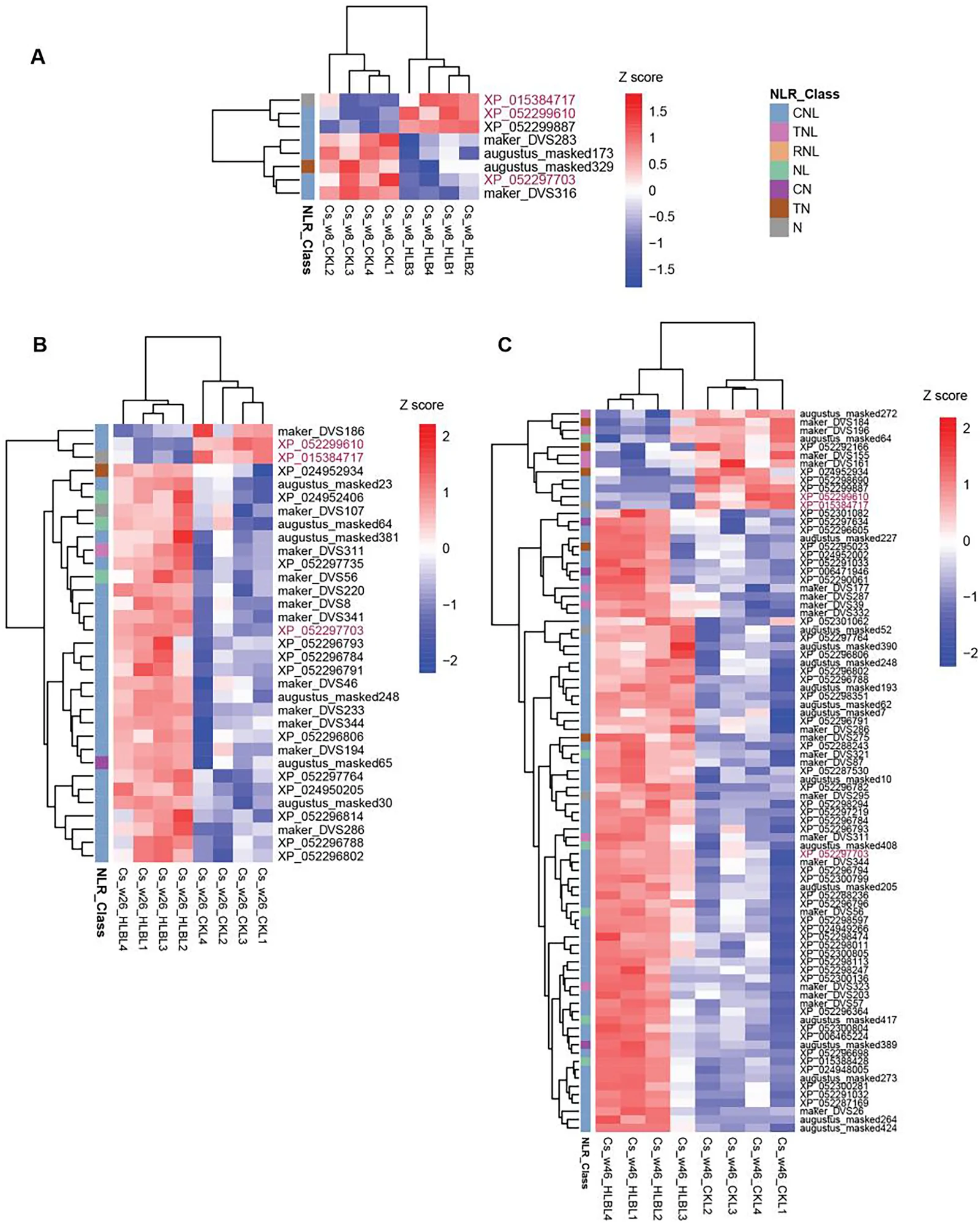
Differentially expressed NLR genes in *C. sinensis* in response to *C*Las infection. **(A)** The expression patterns at 8-week post graft-inoculation with *C*Las (wpg). **(B)** At 26 wpg, more NLR genes were induced. **(C)** At 46 wpg, more NLR genes were upregulated. DEGs found at three time points were indicated in pink.

Mapping the differentially expressed NLRs onto the pan-NLRome orthogroups localized the response to a small number of groups. Seventeen derived from a single core CNL orthogroup, OG0000004, were all upregulated at 26 and/or 46 wpg; one member, XP_052297703, was upregulated at 8, 26, and 46 wpg. OG0000004 was present at markedly higher copy number in *C. sinensis* than in the other species examined, with 26–30 copies compared with six in *S. glutinosa*. Estimates were consistent across independent *C. sinensis* assemblies and cultivars, indicating that the expansion is not an assembly artefact. Two NLRs also responded at three time points: XP_052299610, from the core orthogroup OG0000116, in which most species carry a single copy, and XP_015384717, which retains only the NB-ARC domain and derives from the dispensable orthogroup OG0000364. OG0000004 therefore combines lineage-specific copy-number expansion with the strongest and most sustained transcriptional response to *C*Las, suggesting it as an important CNL orthogroup engaged during *C*Las infection in *C. sinensis*.

### CNGC gene family

Cyclic nucleotide-gated channels (CNGCs) function in multiple pathways of plant development, growth, immunity and environmental adaptation, and mediate the calcium influx required for both Pattern-triggered immunity (PTI) and effector-triggered immunity (ETI) (Tian et al., 2019). We identified 17 CNGCs in *S. glutinosa*. CNGC numbers varied across the genotypes examined, from 10 in *C. australasica* to 25 in *C. hongheensis*.

Phylogenetic analysis of citrus and *Arabidopsis* CNGCs revealed four major groups (I–IV) (**Supplementary Figure S8A**), consistent with previous classifications (Chin et al., 2009), with Group IV further divided into subgroups IV-a to IV-d. The defense-related *Arabidopsis* CNGC2 and CNGC4 fell into Group IV and CNGC11 and CNGC12 in Group II, as previously reported^31^. CNGC19 and CNGC20, also defense-related, formed subgroup IV-a on their own, with no citrus CNGC from the genotypes examined belonging to this clade.

To examine tissue-level expression, we analyzed the CNGCs belonging to pangenome core gene families in the *C. reticulata* tissue panel (Wen et al., 2024). CNGCs in Groups II, IV-c and IV-d shared a similar profile, with high expression in leaves and in the pulp and seeds of mature fruit, while those in Groups III and IV-b showed the opposite pattern (**Supplementary Figure S8B**; **Supplementary Table S10**). Under *C*Las infection (Chin et al., 2020), most differentially expressed CNGCs were downregulated. Induction was limited to few samples in Groups I, II, IV-c and IV-d and showed no consistent pattern (**Supplementary Figure S8C**; **Supplementary Table S11**).

### Pathogenesis-related proteins in citrus and citrus relatives

Pathogenesis-related (PR) proteins accumulate during both PTI and ETI and are usually up-regulated by salicylic acid (Han and Schneiter, 2024). Of the 19 PR families described to date (Sharma et al., 2022), most show antimicrobial activity through various modes of action: PR6, PR12, PR13 and PR14 act as antimicrobial peptides, while others are enzymes, including β-glucanase (PR2), chitinases (PR3, PR4, PR8, PR11), proteinase (PR7), peroxidase (PR9), RNase (PR10), oxalate oxidases (PR15, PR16) and carbohydrate oxidase (PR18).

Seventeen of the 19 *PR* families were identified across the 25 citrus genotypes, and *PR13* and *PR19* were absent from all genomes examined. Total *PR* gene number ranged from 315 in *C. australasica* to 473 in *C. maxima* ‘Wanbaiyou’, with 358 in the *S. glutinosa* collapsed genome assembly (**Figure 5A**; **Supplementary Tables S12, 13**).

**Figure 5:**
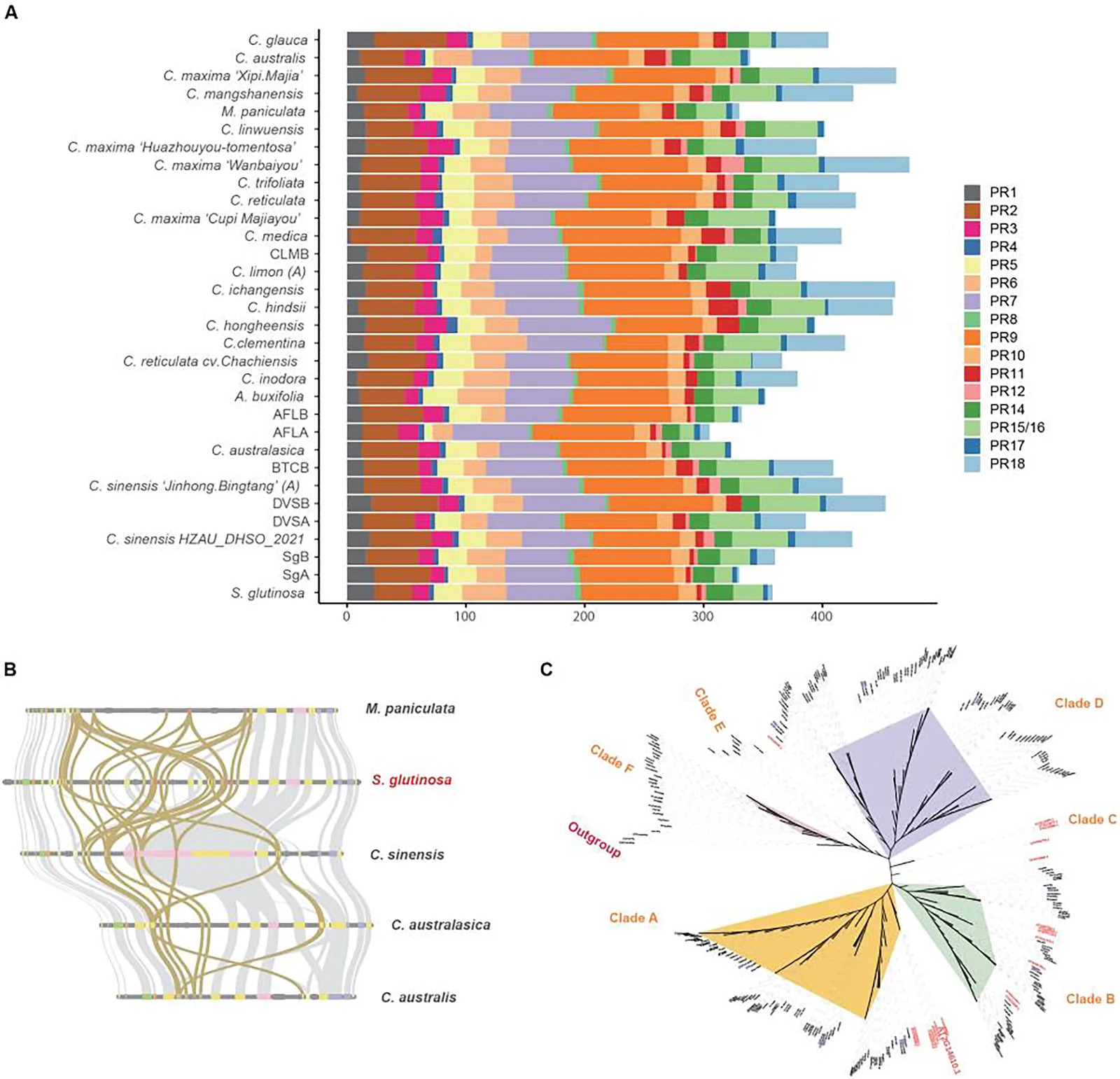
PR proteins in citrus. **(A)** The number of *PR* genes in each genome. **(B)** The genome synteny of *PR1* between *S. glutinosa* and other citrus species and relatives. **(C)** The phylogenetic tree of *PR1* in citrus species and relatives. The *PR1* genes in *S. glutinosa* are shown in purple.

Expansion of *PR15* and *PR16*, and a larger expansion of *PR18*, were found in most citrus genotypes but not in *C. australasica* or *C. australis*. *PR* gene expression was also examined in *C. sinensis* at 26 wpg by *C*Las (**Supplementary Figure S9**). *PR1*, *PR2* and *PR5* were expressed, consistent with canonical salicylic acid-regulated defense responses. *PR18* was also induced, indicating that the response is not restricted to the canonical *PR* gene families.

### Antioxidant enzymes in citrus

ROS production is triggered by both PTI and ETI, and antioxidant enzymes maintain ROS homeostasis to limit oxidative damage.

Antioxidant enzyme gene number ranged from 118 in *C. maxima* ‘Huazhouyou-tomentosa’ to 174 in *C. medica* (**Supplementary Figure S10**). The *S. glutinosa* collapsed assembly encoded 83 peroxidases (POX), 28 glutathione S-transferases (GST), 14 superoxide dismutases (SOD), 6 dehydroascorbate reductases (DHAR), 5 glutathione peroxidases (GPX), 4 glutathione reductases (GR), 3 monodehydroascorbate reductases (MDHAR) and 2 catalases (CAT). *C. sinensis* (Di-Haploid Sweet Orange; HZAU v3.0) contains 75 POX, 29 GST, 14 SOD, 4 DHAR, 4 GPX, 4 GR, 3 MDHAR and 1 CAT (**Supplementary Figure S10**; **Supplementary Table S14**).

### Antimicrobial peptides in *S. glutinosa* and other citrus species

A stable antimicrobial peptide, MaSAMP, of 67 amino acids from the HLB-resistant *C. australasica* was reported to confer HLB resistance by inhibiting *C*Las growth (Huang C. Y. et al., 2021). *S. glutinosa* encodes two MaSAMP homologues of 157 and 112 amino acids, sharing 93.22% and 46.30% identities with MaSAMP, respectively. Two homologues were likewise present in each of the remaining 23 genotypes, with identities ranging from 88.71% to 96.77% for the longer copy and 44.44% to 52.38% for the shorter; *C. glauca* carried three, two of which shared 95% identity. Structure prediction indicated that both *S. glutinosa* peptides contain α-helical and β-sheet elements (**Supplementary Figure S11**).

We then predicted AMPs genome-wide in the susceptible *C. sinensis* and the resistant *C. australasica*, *S. glutinosa* and *C. australis* using a deep learning method. The numbers of predicted AMPs were 177, 76, 40 and 37, respectively (**Supplementary Table S15**), of which 88, 39, 27 and 16 were predicted to have antibacterial activity.

In *S. glutinosa*, predicted AMPs ranged from 6 to 184 amino acids. Twenty-eight had at least one InterPro hit (*P* < 0.0005), including six defensin-like proteins and four histone H4 proteins. Histones have antimicrobial activity in the extracellular environment (Parseghian and Luhrs, 2006), with H2A- and H4-derived peptides from silver pomfret shown to act against Gram-negative bacteria (Huang et al., 2024). AMPs were unevenly distributed across the nine pseudochromosomes, with 20% clustered on chromosome 7 (**Supplementary Figure S12**), and 25% were species-specific.

Defensin-like proteins were also recovered in the other species, with seven in *C. australasica*, eight in *C. australis* and eleven in *C. sinensis*, six of the latter carrying a predicted signal peptide. Histone H4-derived AMPs were predicated in *C. australasica* (three), *C. australis* (four) and *C. sinensis* (five).

Because many predicted AMPs could not be assigned to known domains, we constructed a phylogeny of 309 non-redundant sequences, which resolved into six clades (**Figure 6**). Clade A was the largest and contained the defensin-like proteins (74 AMPs: 11 from *S. glutinosa*, 15 from *C. australis*, 16 from *C. australasica* and 32 from *C. sinensis*). The histone H4-derived AMPs formed a subclade within Clade C; 14 of these 15 sequences were 103 amino acids and identical, as expected for histone H4, with the exception of the 58-amino-acid Cs_ont_3g028380 (**Supplementary Figure S13**). Sequences in the remaining clades could not be assigned to known superfamilies.

**Figure 6:**
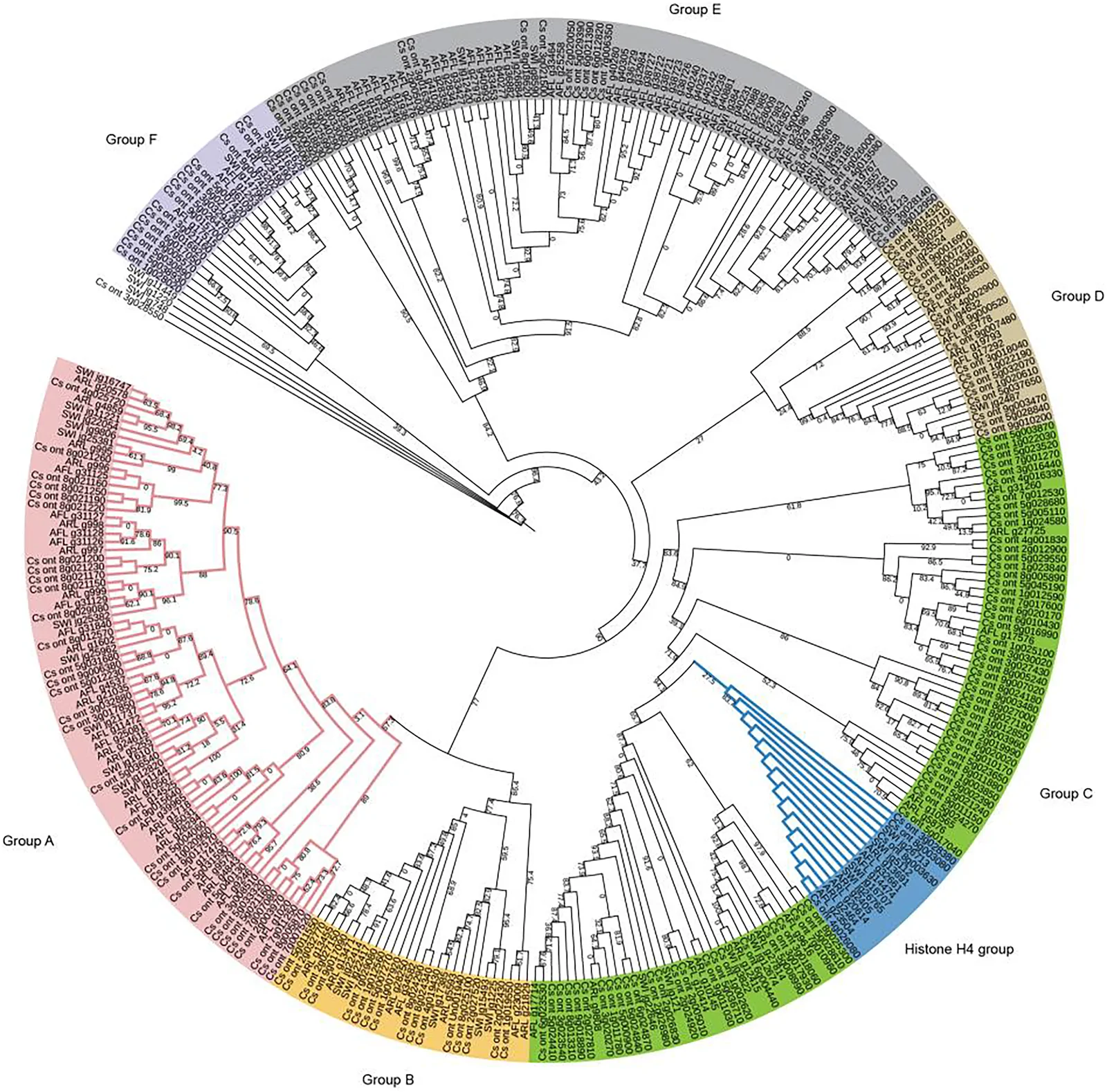
Phylogenetic analysis of predicted antimicrobial peptides in *C. sinensis, C*. *australasica, S. glutinosa* and *C. australis*.

## Discussion

In this study, we provide a high-quality haplotype- resolved chromosome- level reference genome of the HLB-resistant citrus relative *S. glutinosa*. We have analyzed species-specific genes and immune related genes of *S. glutinosa* that may contribute to its resistance to HLB.

### Species-specific genes and candidate resistance-associated pathways

HLB severity varies widely across citrus species, wild relatives and other Rutaceae, and tolerance or resistance has been reported in *A. buxifolia*, *C. glauca*, *C. australasica*, *C. australis*, *C. inodora*, *C. ichangensis*, *C. trifoliata* and *M. paniculata* (Alves et al., 2020; Ramadugu et al. (2016b). We identified 1,089 genes specific to *S. glutinosa*, enriched for the cullin-RING ubiquitin ligase (CRL) complex. CRLs coordinate SA-induced protein ubiquitination and contribute to plant immune signaling (Furniss and Spoel, 2015). Within SA signaling, NPR1 forms a functional NPR1–CRL3 adaptor complex that contributes to protein homeostasis and cell survival (Zavaliev et al., 2020), and E3 ubiquitin ligases more broadly are implicated in biotic and abiotic stress tolerance and DNA repair (Mazzucotelli et al., 2006). Additionally, *S. glutinosa* specific genes include those encoding 4-coumarate--CoA ligase, which is involved in flavonoid biosynthesis and JA signaling (Alariqi et al., 2023); and chalcone/stilbene synthases, which are involved in production of phytoalexins and flavonoids (Kodan et al., 2002). Many flavonoids have antimicrobial activities (Hijaz et al., 2020). JA signaling pathway was reported to be involved in citrus resistance to HLB (Zhao et al., 2025).

Genes specific to the resistant group were separately enriched for CoA-ligase activity and auxin transport, and many were annotated as auxin response factors (ARFs), which have been reported to mediate auxin action in tomato responses to biotic and abiotic stress (Bouzroud et al., 2018). Intriguingly, LYK4 (lysM domain receptor-like kinase 4), which is important for detection of chitin to activate immune responses (Wan et al., 2012), was only identified in *C. glauca, C. australis*, and *C. inodora* of the HLB-resistant group.

### NLR repertoire size and transcriptional induction are not sufficient for resistance

NLR genes are crucial for detecting invading pathogens and initiating immune signaling (van Wersch et al., 2020). To catalogue the NLR repertoire across the genotypes examined, we constructed a pan-NLRome representing all NLR genes identified across the 29 accessions. HLB-susceptible genotypes generally carried more NLR copies than HLB- resistant or tolerant genotypes, indicating that a larger NLR repertoire does not correspond to stronger resistance or tolerance to HLB. It remains to be determined whether the differences in NLRs contribute to the proposed model of HLB being a pathogen-triggered immune disease (Huang et al., 2026; Ma et al., 2022). Furthermore, clustering accessions by NLR orthogroup composition recovered species-level structure but did not separate HLB-resistant from HLB- susceptible taxa, and TNL and TNLJ expansions were found not only in HLB-susceptible species but also in the HLB-resistant *C. australasica* and *C. australis*. Resistance is therefore not associated with a consistent reduction in any single NLR subclass, and the contraction in overall repertoire size does not extend uniformly to individual families.

Transcriptional responses pointed to CNLs as an important NLR group responding to infection. Paradoxically, the HLB-susceptible *C. sinensis* carried more copies of OG0000004 than the HLB- resistant *S. glutinosa*, and all these copies were upregulated upon infection, indicating that expansion of this orthogroup does not confer effective resistance, but may contribute to the chronic immune responses including ROS production and phloem callose deposition triggered by *C*Las (Huang et al., 2026; Ma et al., 2022). Functional characterization of individual members of this orthogroup will be needed to resolve their roles in the HLB resistance and disease development.

### CNGCs

CNGCs are an essential component of plant innate immunity and interact with many receptor kinases (Dietrich et al., 2020). For instance, CNGC2 and CNGC4 are critical for PAMP-induced calcium signaling (Tian et al., 2019). Under *C*Las infection, most differentially expressed citrus *CNGCs* were downregulated rather than induced. Because CNGCs mediate the calcium influx that initiates immune signaling, reduced transcript levels during infection may limit the calcium-dependent responses available to the host, although whether this reflects a host response or interference by the pathogen cannot be resolved from these data.

### PR proteins and systemic acquired resistance

Systemic acquired resistance (SAR) is a long-lasting form of immunity triggered by an initial pathogen challenge, in which PR1, SA and NON-EXPRESSOR OF PR GENES 1 (NPR1) are key components, and *PR* gene expression is induced through the NPR1-dependent SA pathway (Durrant and Dong, 2004). The 19 PR families include pathogenesis-related peptides and AMPs such as plant defensins (PR12) and thionins (PR13) (Sels et al., 2008), and PR proteins contribute to biotic stress responses in plants including rice (Mitsuhara et al., 2008), poplar (Wang et al., 2023) and citrus (Wu et al., 2018). In *S. glutinosa*, the PR1 family was expanded compared to other citrus genotypes, with proximal duplication contributing to the increased copy number. PR1 has strong antimicrobial activity against diverse plant pathogens including oomycetes, fungi, and bacteria (Guo et al., 2022; Niderman et al., 1995; Rauscher et al., 1999). In addition, PR1 was reported to be proteolytically cleaved to release a C‐terminal CAPE1 peptide to activate immune responses (Chen et al., 2014). Thus, the expanded PR1 repertoire in *S. glutinosa* may provide greater capacity for PR1-mediated defense against *C*Las.

### Antimicrobial peptides

MaSAMP from Australian finger lime (*C. australasica*) was recently reported to inhibit *C*Las growth and induce PR gene expression (Huang C. Y. et al., 2021), suggesting a possible route to controlling the pathogen. More AMPs were identified in *C. australasica* later on (Younas et al., 2025). AMP-encoding genes occur widely across prokaryotic and eukaryotic genomes, and AMPs are often expressed as inactive precursors comprising a signal peptide that drives secretion, a pro-peptide region, and a mature peptide released by proteolysis (Rončević et al., 2019). In plants they are classified by sequence homology into families including defensins, thionins, lipid transfer proteins, cyclotides, snakins and hevein-like proteins (Nawrot et al., 2014), and are frequently upregulated by SA, JA and ethylene (Campos et al., 2018).

*S. glutinosa* encodes two homologues of MaSAMP, both predicted to combine α-helical and β-sheet elements, together with 40 other AMPs predicted by a deep learning method, including defensin-like and histone H4 proteins. Those assigned to known families were conserved between *S. glutinosa* and citrus, but a quarter of the *S. glutinosa* AMPs were species-specific, and many predicted AMPs across the species examined could not be assigned by either database search or phylogenetic analysis. *C. sinensis* contributed a relatively large number of short sequences to the defensin-like clade, but these lacked a complete defensin domain and a signal peptide region, so the higher sequence count in this species does not correspond to a larger amount of functional defensins.

AMP function is closely tied to structure. In the *C. australasica* peptide, the second of two short α-helices forms pore-like oligomers that insert into the bacterial membrane and cause cytoplasmic leakage and cell lysis (Huang C. Y. et al., 2021). Structure prediction and direct functional testing will therefore be needed to determine whether the lineage-specific AMPs of *S. glutinosa* contribute to its resistance to *C*Las.

## Conclusions

Overall, this study generated a high-quality haplotype-resolved chromosome-level reference genome for *S. glutinosa* and provides valuable genomic resources for investigating HLB resistance in citrus species and its relatives. Comparative analysis of defense-associated gene families, including NLRs, PRs, and AMPs, identified candidate genomic features that may contribute to the distinct HLB response of *S. glutinosa*. These resources and findings will support future functional studies of HLB resistance mechanisms and inform citrus breeding efforts.

## Materials and methods

### Plant materials and genome sequencing

*S. glutinosa* plants were maintained in a greenhouse in Lake Alfred, Florida, USA with natural light and without temperature control. *S. glutinosa* plants were grown from seed. Genomic DNA was isolated from the *S. glutinosa* leaf tissue using a modified version of the CTAB method (Wang et al., 2006). Briefly, leaf tissue was flash frozen in liquid nitrogen and homogenized using a TissueLyser II (Qiagen, Valencia, CA, USA). The powdered tissue was resuspended in 650 uL of CTAB extraction buffer (2% CTAB, 1.4 M NaCl, 0.02 M EDTA (Ethylenediaminetetraacetic acid), 0.1 M Tris HCl, 1% w/v PVP40 (Polyvinylpyrrolidone)) and incubated at 65˚C for 30 min. Beta-mercaptoethanol was added to the CTAB extraction buffer and prior to extracting with chloroform, the mixture was first extracted with an equal volume of phenol-chloroform. An equal volume of chloroform was added, the tubes were mixed, centrifuged, and the aqueous layer was transferred to a new tube with an equal volume of isopropanol. After pelleting the precipitated nucleic acid, the pellet was washed with 75% ethanol, dried, and resuspended in 50 μL of water. The DNA concentration was measured using Synergy LX plate reader (Agilent, Santa Clara, CA, USA). Genomic DNA was used to construct 15-kb libraries for PacBio HiFi read production using a standard protocol provided by PacBio (Pacific Biosciences, USA). HiFi long-read sequencing was performed using PacBio Sequel II platform by BGI (BGI, Shenzhen, China). Short-read sequencing was conducted using Illumina platform by Novogene (Novogene, Beijing, China), generating 150-bp paired end reads. Hi-C libraries construction and sequencing were completed by Novogene and BGI using 150-bp paired-end strategy, and a total of ~28.8 Gb and ~141 Gb Hi-C clean reads were generated, respectively.

### Genome assembly

To obtain the collapsed genome assembly, HiFi clean reads were assembled into the initial assembly using hifiasm (v0.19.8-r603) (Cheng et al., 2021). Purged_dup (v1.01) was used to remove duplications (Guan et al., 2020). Hi-C reads were used to scaffold the contig assembly into chromosome-scale level. The read alignment process was performed via juicer (v1.60) (Durand et al., 2016b). Then 3D-DNA (v190716) (Dudchenko et al., 2017) with the parameter “-r 0” was used to construct the chromosome- level assembly. Juicebox (Durand et al., 2016a) was used to manually adjust the assembly to 9 pseudochromosomes.

Both HiFi clean reads and Hi-C reads were used to assemble the two sets of phased contig level assemblies (haplotype A and haplotype B) via hifiasm (v0.19.8-r603) Hi-C integrated method (Cheng et al., 2021). The contig level haplotype assemblies were then scaffolded into chromosome-level using 3D-DNA(v190716) pipeline (Dudchenko et al., 2017). The quality of the genome assembly was evaluated by using BUSCO (v5.3.0, embryophyte_db, n=1640) (Manni et al., 2021). Illumina short reads were mapped to the collapsed and phased genomes using BWA (Li and Durbin, 2010).

### Genome annotation

Repeat elements were first identified by LTR_FINDER (Xu and Wang, 2007), RepeatModeler (https://repeatmasker.org/RepeatModeler/) and EDTA (Ou et al., 2019). All the results were processed as a custom library input to RepeatMasker (v4.1.1) (http://www.repeatmasker.org), which eventually predicted the repeat sequences and produced the soft masked genome. An integrated method using RNA-seq and related-species protein data as evidence was adopted to perform gene prediction by BRAKER2 (Brůna et al., 2021). RNA-seq reads of *S. glutinosa* were used for both genome annotation and NLR expression analysis. *S. glutinosa* RNA extraction and sequencing were conducted as described previously (Hendrich et al., 2025). For genome annotation, quality-filtered reads were mapped to the chromosome-level *S. glutinosa* genome assembly and incorporated as transcript evidence to support protein-coding gene prediction, including exon-intron boundary refinement and transcript structure validation. The same RNA-seq dataset was further used to estimate expression levels of annotated *S. glutinosa* NLR genes.

BUSCO analysis was used to check for the gene set completeness of the assemblies (Manni et al., 2021). The re-annotation of *C. inodora*, and *C. glauca* was conducted using the same method. Gene functional annotation was performed using eggNOG-mapper v2 (Cantalapiedra et al., 2021) and InterProScan (Jones et al., 2014), and the predicted protein sequences were searched against the public databases such as KEGG (Kanehisa et al., 2017), UniProt (The UniProt Consortium, 2021), and NR (https://www.ncbi.nlm.nih.gov/) using BlastP (Camacho et al., 2009). The program iTAK (v1.7) (Zheng et al., 2016) was used to identify transcription factors in *S. glutinosa* genome.

### Gene family analysis

We used Orthofinder (v2.5.5) (Emms and Kelly, 2019) to identify shared gene families in citrus and citrus relatives, and species-specific genes in *S. glutinosa*. Only the longest mRNA of each gene was kept for the downstream analysis. The 23 citrus and citrus relative genomes include *C. ichangensis*, *C. maxima* ‘Wanbaiyou’, *C. medica*, *A. buxifolia* (Wang et al., 2017), *C. australasica* (Nakandala et al., 2024b), *C. australis* (Nakandala et al., 2023), *C. clementina* (Wu et al., 2014), *C. inodora* (Nakandala et al., 2024a), *C. glauca* (Singh et al., 2024), *C. limon* (Bao et al., 2023), *C. hindsii* (Peng et al., 2020), *C. hongheensis*, *C. linwuensis*, *C. mangshanensis*, *C. maxima* ‘Xipi Majia’(Huang et al., 2023b), *C. maxima* ‘Cupi Majiayou’ (Lu et al., 2022), *C. maxima* ‘Huazhouyou-tomentosa’ (Zheng et al., 2023), *C. reticulata* (wild mandarin) (Wang et al., 2018), *C. reticulata* cv. Chachiensis (Wen et al., 2024), *C. sinensis* (Di-Haploid Sweet Orange; HZAU v3.0) (Wang et al., 2021), *C. sinensis* ‘Jinhong Bingtang’ (Wang et al., 2024a), *C. sinensis* cv. Valencia (DVS) (Wu et al., 2022), *C. trifoliata* (Huang, Y. et al., 2021), *M. paniculata* (Yang et al., 2023). The protein sequences of these genomes were downloaded from CPDB (http://citrus.hzau.edu.cn/), NCBI (https://www.ncbi.nlm.nih.gov/) and the National Genomics Data Center (https://ngdc.cncb.ac.cn/). Functional enrichment analysis was performed by clusterProfiler 4.0. CAFE5 (Mendes et al., 2021) was used to analyze the expansion and contraction of the gene families.

### Transcriptome analysis

RNA-seq data were retrieved from the study CRA015571 from GSA and PRJNA417324 from NCBI. STAR (v2.7.9a) (Dobin et al., 2013) was used to perform the mapping steps with the 2-pass mode, and R package DESeq2 (Love et al., 2014) was used for read count normalization and differential gene expression analysis.

### Genome synteny and whole-genome duplication analysis

The genome synteny between the citrus genomes was analyzed by JCVI (Tang et al., 2024). JCVI was also used to infer whole-genome duplication (WGD) events by intra-genome analysis and for gene synteny analysis. SyRI (Goel et al., 2019) was used to detect structure variations between different genome assemblies. DupGen_finder (Qiao et al., 2019) was used to classify the type of gene duplication.

### Identification of CNGC proteins, PR proteins, antioxidant enzymes and antimicrobial peptides

*A. thaliana* CNGC protein sequences were used as reference sequences to identify CNGC homologs in 25 citrus accessions. The *Arabidopsis* CNGC sequences were aligned and used to build a hidden Markov model. The resulting HMM profile was searched against the predicted citrus protein set to identify candidate CNGC proteins. Candidate sequences were further examined based on conserved CNGC-related domains and sequence features. Similarly, to identify PR proteins, the protein sequences were first searched against the PR reference sequences with hmmsearch by HMMER (v3.4; Aug 2023) (http://hmmer.org/) and confirmed by the existence of the corresponded conserved domain. The identification of antioxidant enzymes was using sequences of *A. thaliana* and *Oryza sativa* from UniProt as references and then confirmed by the conserved domain.

MAFFT (v7.490) (Katoh and Standley, 2013) was used to construct the alignment matrix and the tool trimAl (Capella-Gutiérrez et al., 2009) was used for trimming the alignments with the parameter “-gt 0.95 -cons 20 -w 3”, and IQ-TREE (Nguyen et al., 2015) was used to construct the phylogenetic tree with 1000 bootstraps with the parameters “-alrt 1000 -B 1000”.

The identification of AMPs was performed using the deep learning based software iAMPCN (Xu et al., 2023). Amino acid sequences with lengths of more than 300 aa were filtered before prediction, and the sequences with in-frame stop codon were also dropped. All AMPs were searched against InterPro (Paysan-Lafosse et al., 2023) for classification. The alignment visualization was conducted using Jalview (Waterhouse et al., 2009). The AMPs structure models were predicted by Alphafold 3 (Abramson et al., 2024). The online tool MG2C (Chao et al., 2021) (http://mg2c.iask.in/mg2c_v2.1/) was used to visualize the distribution of AMPs.

For phylogenetic analysis of AMPs and PR1 proteins, MUSCLE (Edgar, 2004) and IQ-TREE (Nguyen et al., 2015) were used to perform the sequence alignment step and construct the phylogenetic tree with 1000 bootstraps, respectively.

### Construction of the citrus NLRome

To comprehensively identify NLR genes across the citrus species, we used a two-step strategy. First, an annotation-based pipeline was applied to predicted protein sets derived from existing genome annotations. For each genome, only the longest transcript isoform was retained and translated into the corresponding protein sequence. NLR candidates were identified using RGAugury pipeline (Li et al., 2016), NLRtracker (Kourelis et al., 2021), and Resistify (Smith et al., 2025) which use the machine learning method to detect Nod-like receptors (Martin et al., 2022). Only proteins detected by all three pipelines were retained for downstream analyses. This first step provided a conservative NLR set based on existing gene models.

Second, a genome-guided NLR reannotation pipeline was performed to recover NLR loci that may have been missed or fragmented in standard genome annotations. Genome assemblies were soft-masked using EDTA (Ou et al., 2019). NLR-Annotator (Steuernagel et al., 2020) was then applied to soft-masked genomes to identify candidate NLR loci, and 2 kb flanking sequences were extracted around each candidate locus. These regions were subsequently annotated using MAKER2 (Holt and Yandell, 2011), with species-specific NLR proteins derived from existing genome annotations as homology evidence, supplemented by previously identified NLRs from related genera (Wang et al., 2026). This genome-guided pipeline substantially expanded the NLR repertoire compared to standard genome annotations alone, consistent with the known tendency of LRR domain sequences to be misannotated as repetitive elements during conventional genome annotation pipelines. The two resulting NLR protein sets were mapped back to their respective genomes using miniport (Li, 2023) to unify genomic coordinates. Redundant entries identified by both pipelines were resolved by retaining the longest protein model. Final NLR classification was performed using Resistify (Smith et al., 2025) without CoCoNat (Madeo et al., 2023), because CoCoNat produced fragmented CC-domain predictions that were inconsistent with HMMER-based domain annotations. Additional genomes included in this analysis were *C. sinensis* ‘SWO-M’ and ‘SWO-P’ (Liu S. et al., 2025), *C. sinensis* ‘Parent Washington navel’ (PWNA and PWNB) (de Dios et al., 2026),*C. medica* (CITME), *C. micrantha* (CITMI) *C. reticulata* (Cleopatra mandarin) (CITRE) (Droc et al., 2026), two haplotypes of *C. garrawayi* (MWLA and MWLB), two haplotypes of *C. inodora* (AWLA and AWLB), two haplotypes of *C. glauca* (ADLA and ADLB).

The motif-based approach recovered the majority of CNL and TNL candidates with little dependence on gene prediction completeness, but identified few or no RNLs, consistent with previous reports that current motif-based tools are less suitable for this subclass (Wang et al., 2026). RNL annotation therefore relied on the standard pipeline.

The curated NLR protein sets from 29 citrus and citrus-related accessions were used to construct a pan-citrus NLRome. NLR proteins were clustered into orthogroups using OrthoFinder. For accessions represented by two phased haplotypes, only haplotype A was retained in the OrthoFinder analysis to minimize redundant allelic representation and avoid overestimating NLR family size. The resulting orthogroups were used to characterize core, dispensable, and accession-enriched NLR groups across the pan-citrus dataset.

## Data Availability

The raw sequencing data and genome assembly data generated for *Swinglea glutinosa* have been deposited in NCBI under BioProject PRJNA1223947. The genome annotations, defense-related gene family annotation results, orthogroup tables, and NLR annotation and pan-NLRome results used in this study are available through Zenodo at 10.5281/zenodo.20113701.
