# Supplementary material for: The haplotype-resolved genome of Swinglea glutinosa and pangenome analysis provide insight into resistance to citrus Huanglongbing
Source: Front Plant Sci. 2026 Oct 14;17:1988077. doi: 10.3389/fpls.2026.1988077 (PMC13713493; doi:10.3389/fpls.2026.1988077)
Supplement: Supplementary file 1 [file DataSheet1.pdf]

A

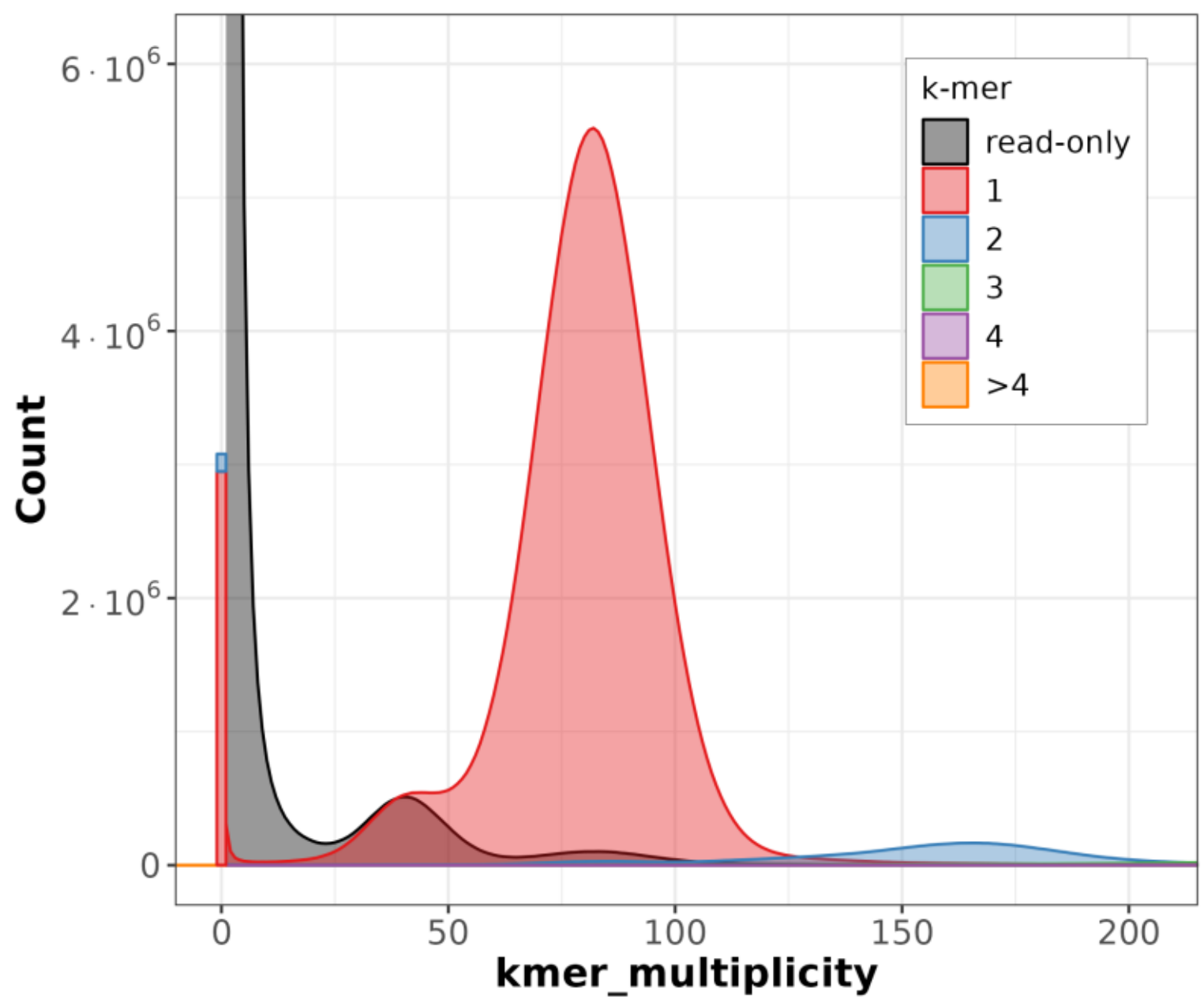

B

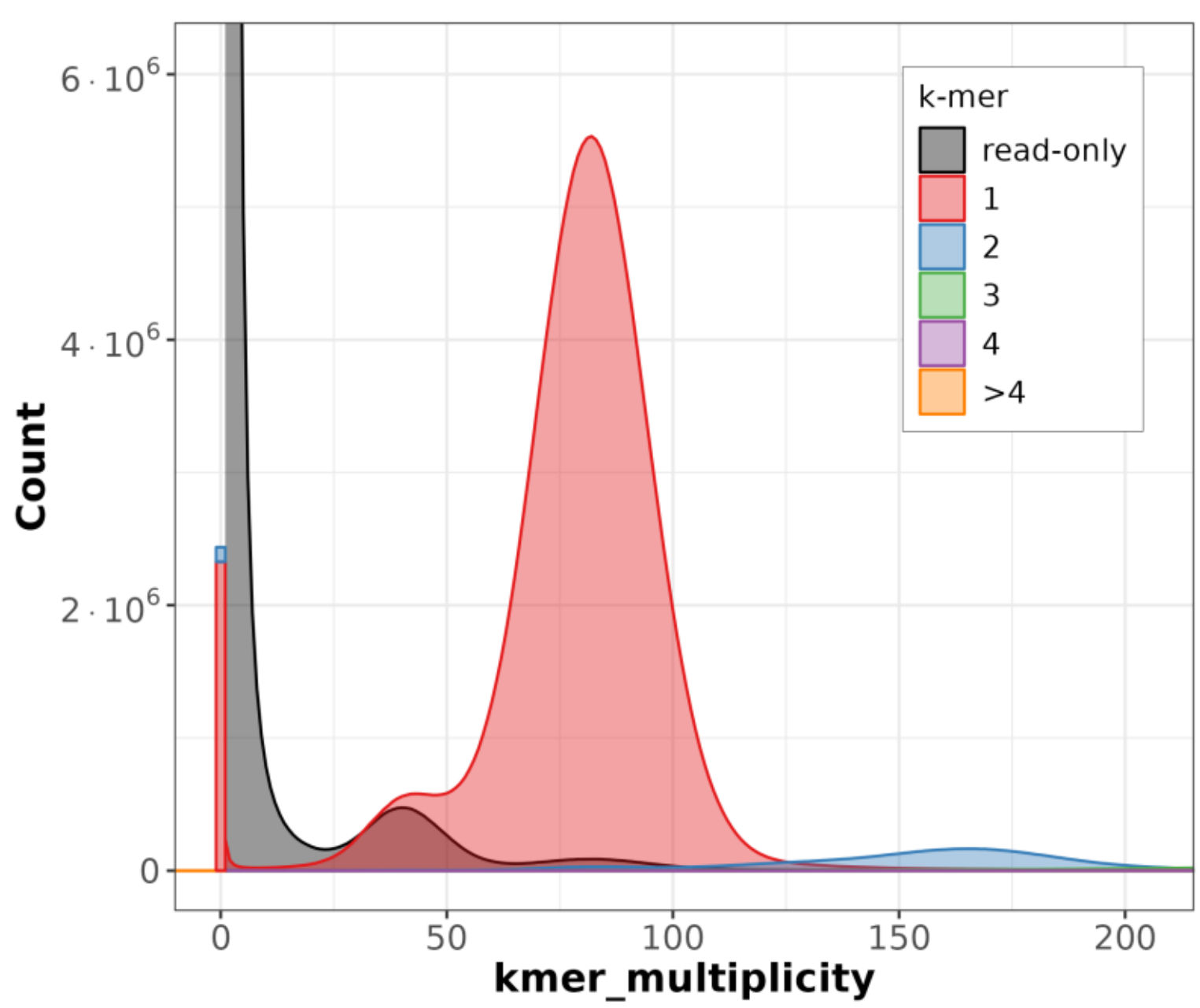

Figure S1. K-mer spectrum analysis of the two haplotype assemblies. A, haplotype A assembly. **B,** haplotype B assembly.

A

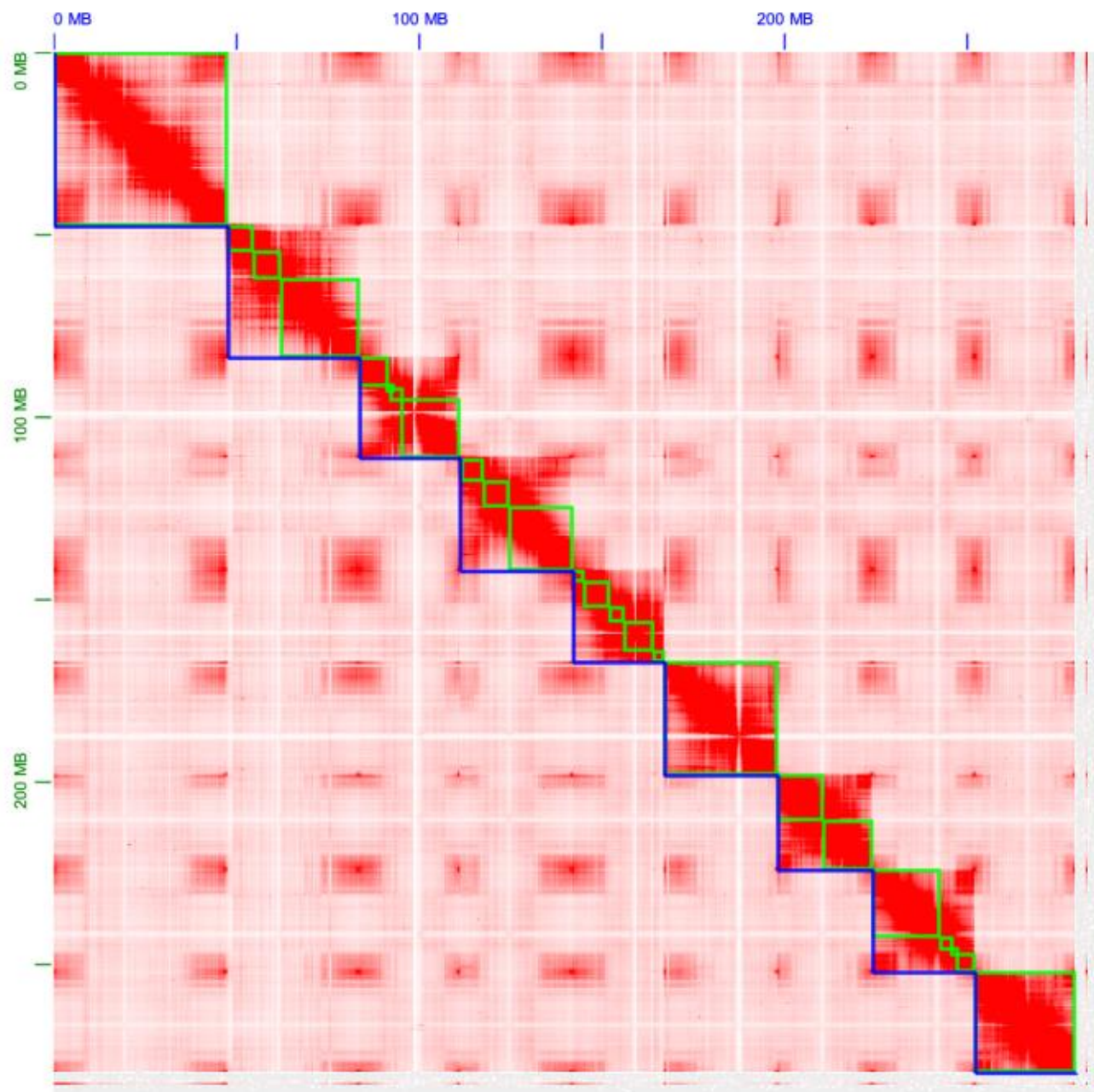

B

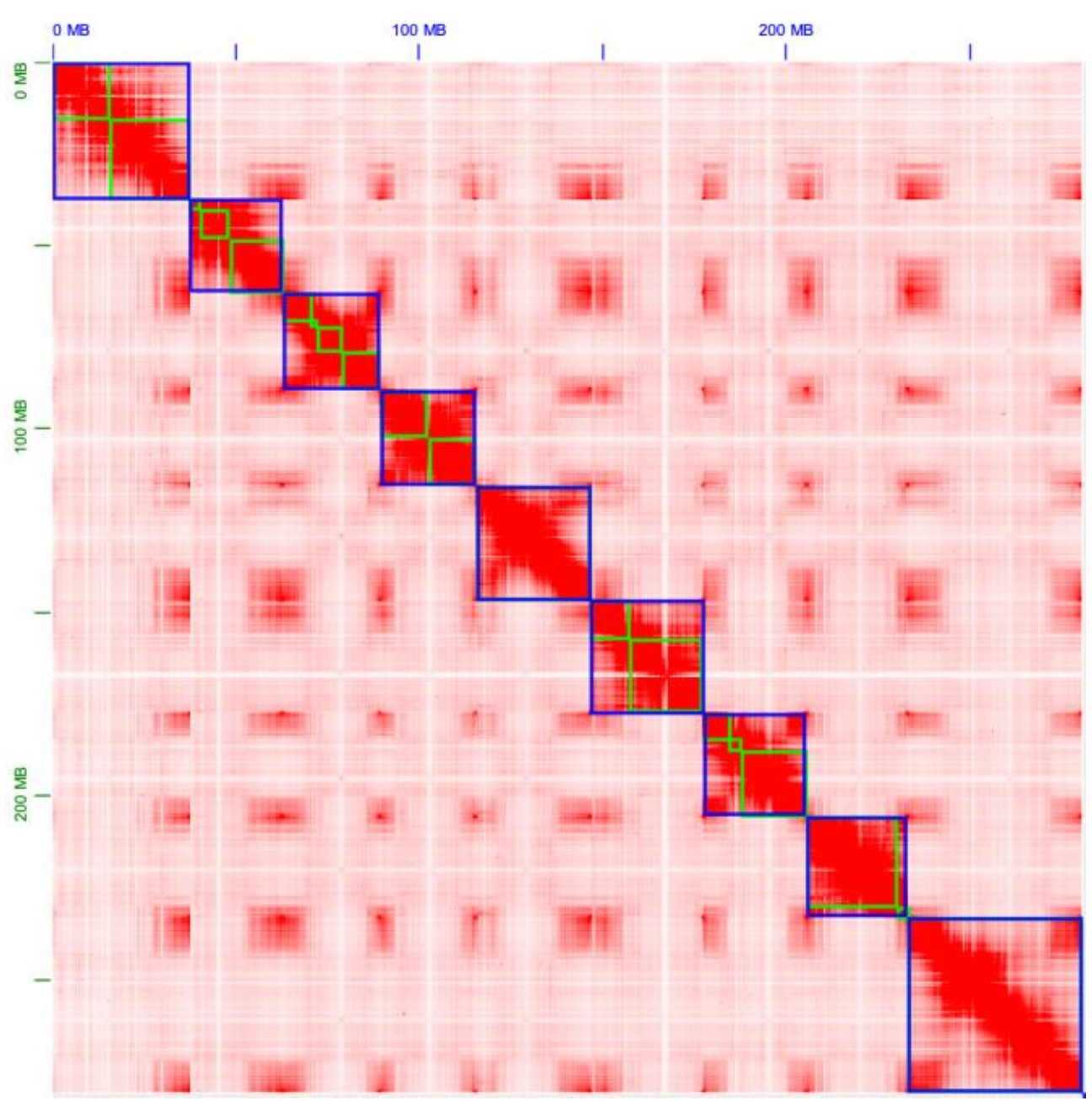

Figure S2. Hi-C contact matrix visualization of the two haplotype genome assemblies. **A,** haplotype A assembly. **B,** haplotype B assembly.

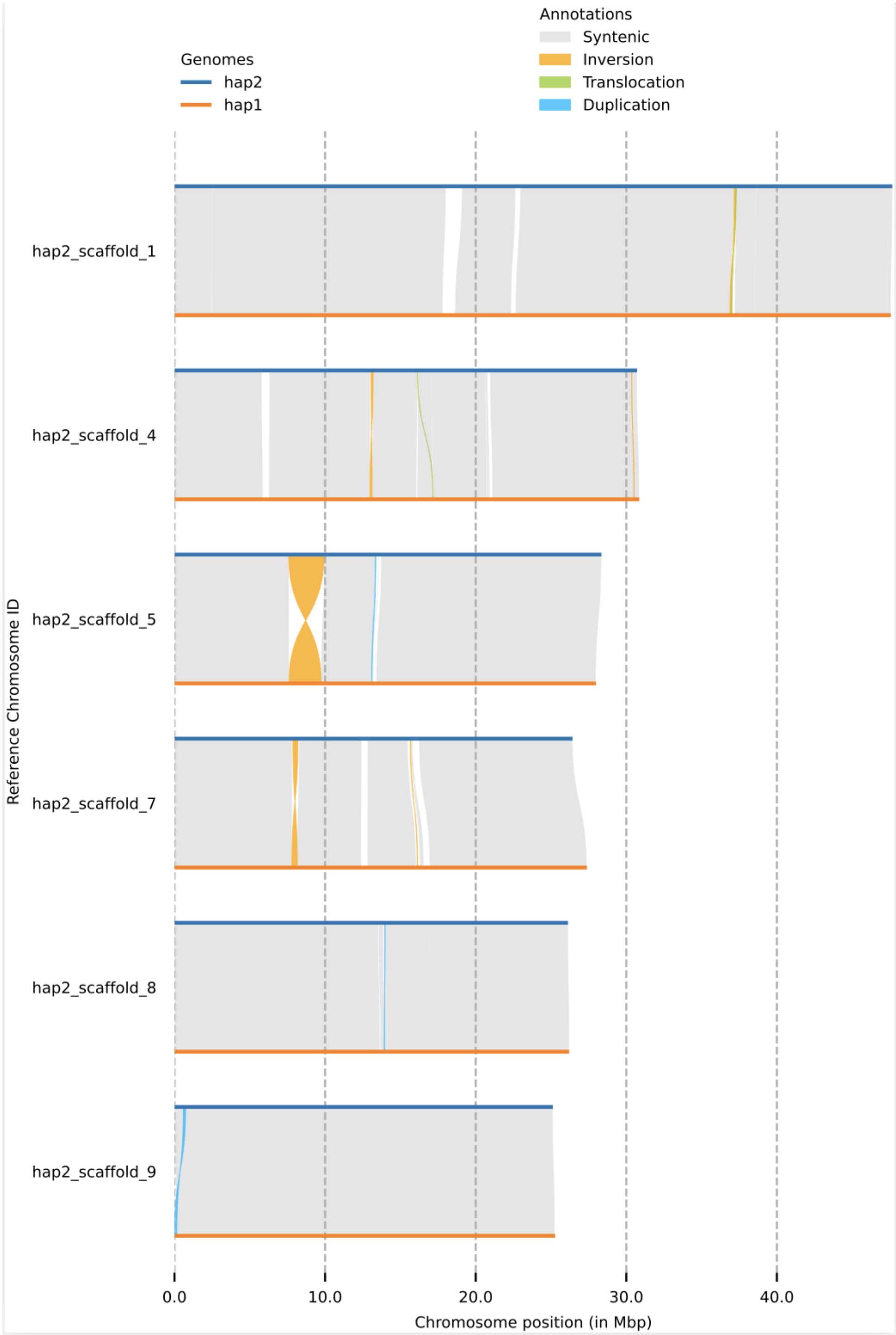

Figure S3. The structure variation in the two haplotype genome assemblies of *S. glutinosa*.The large chromosoal inversion is located on chromosome 5.

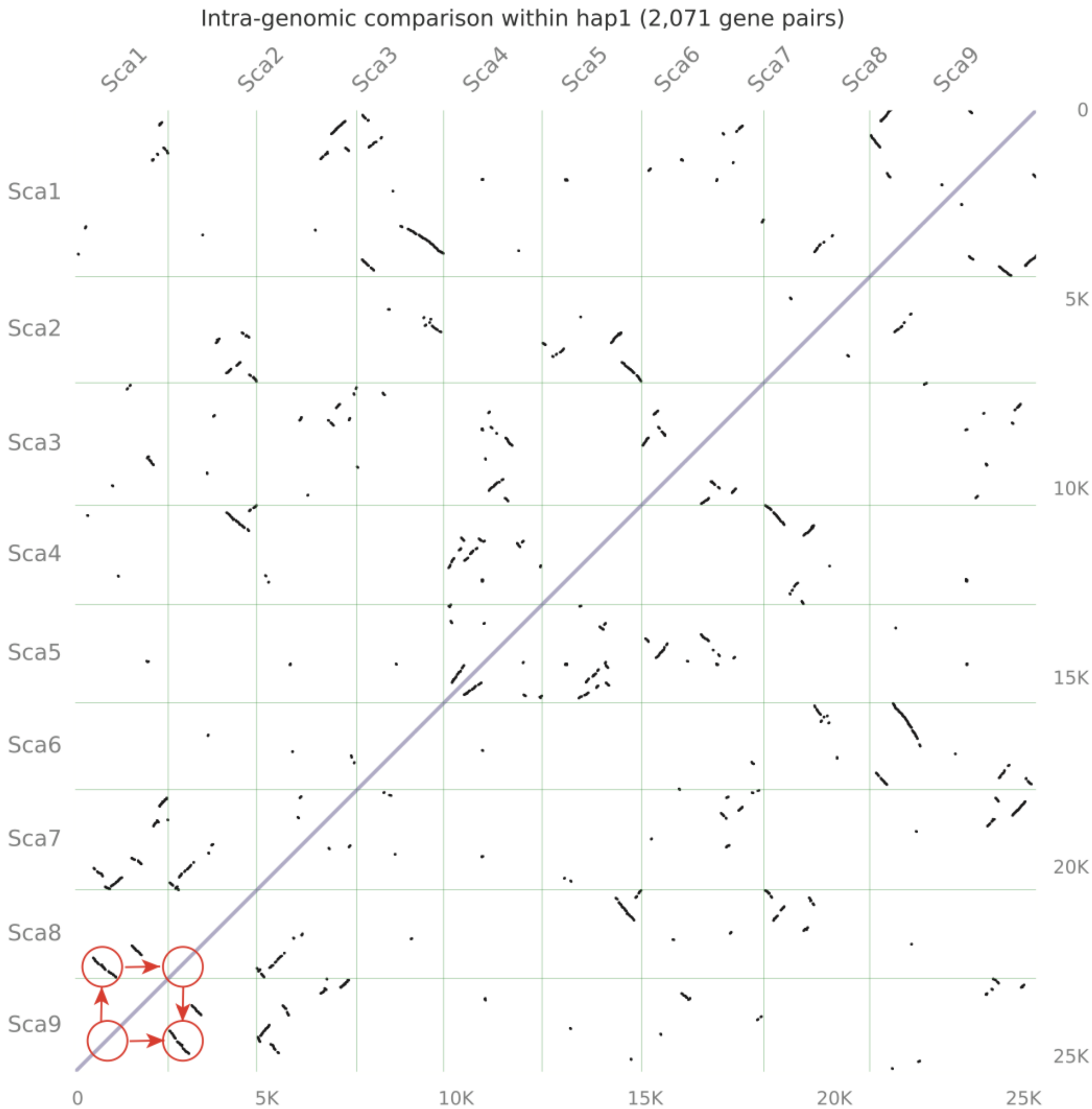

Figure S4. Dot plot of paralogs in the genome of *S. glutinosa*. The red circles indicate that *S. glutinosa* only underwent an ancient WGD event (1–2 chromosomal relationships).

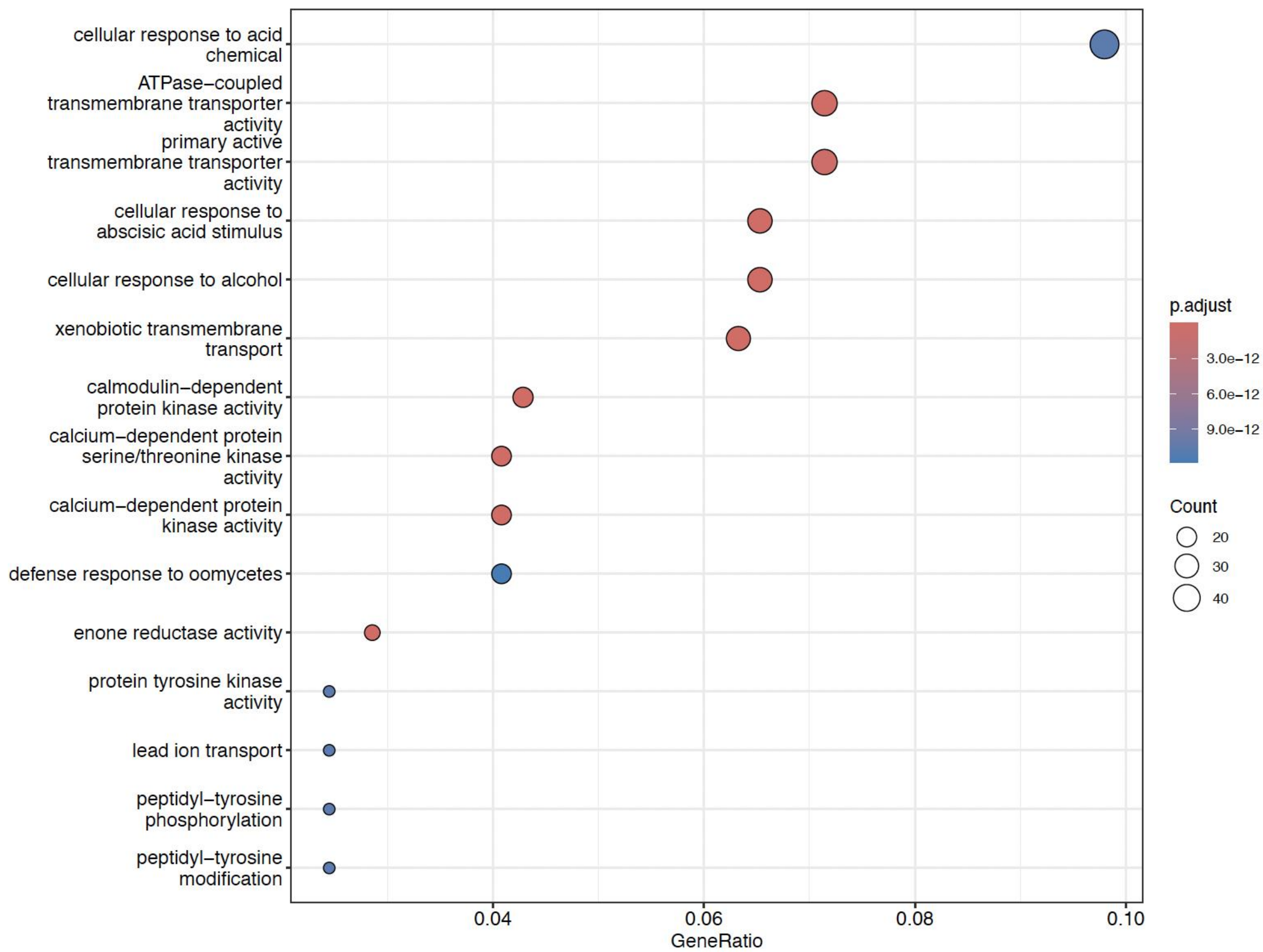

Figure S5. The GO enrichment analysis of *S. glutinosa* expanded gene families.

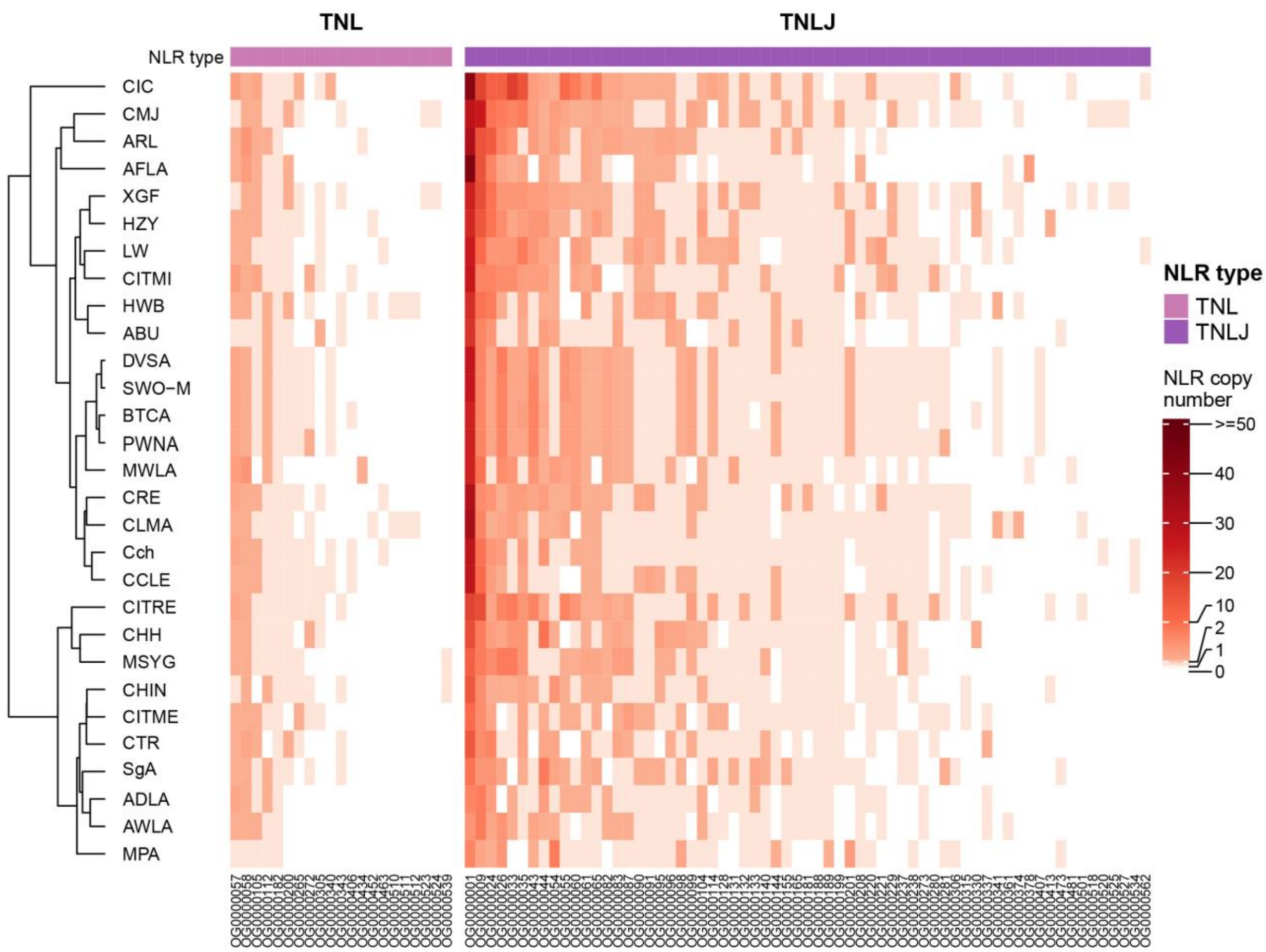

Figure S6. The heatmap of citrus TNL gene families. CIC, *C. ichangensis*; CMJ, *C. maxima* 'Cupi Majiayou'; ARL, *C. australis*; AFLA, *C. australasica* haplotype A; XGF, *C. maxima* 'Xipi Majia'; HZY, *C. maxima* 'Huazhouyou-tomentosa'; LW, *C. linwuensis*; CITMI, *C. micrantha* ; HWB, *C. maxima* 'Wanbaiyou'; ABU, *A. buxifolia*; DVSA, *C. sinensis* cv. Valencia haplotype A; SWO-M, *C. sinensis* (mandarin haplotype); BTCA, *C. sinensis* 'Jinhong Bingtang' haplotype A; PWNA, *C. sinensis* 'Parent Washington navel' haplotype A; MWLA, *C. garrawayi* haplytype A; CRE, *C. reticulata* (wild mandarin); CLMA, *C.limon* haplotype A; Cch, *C. reticulata* cv. Chachiensis; CCLE, *C. clementina*; CITRE, *C. reticulata* (Cleopatra mandarin); CHH, *C. hongheensis*; MSYG, *C. mangshanensis*; CHIN, *C. hindsii*; CITME, *C. medica* ; CTR, *C. trifoliata*; Sg, *S. glutinosa*; SgA, *S . glutinosa* haplotype A; ADLA, *C. glauca* haplotype A; AWLA, *C. inodora* haplotype A; MPA, *M. paniculata*.

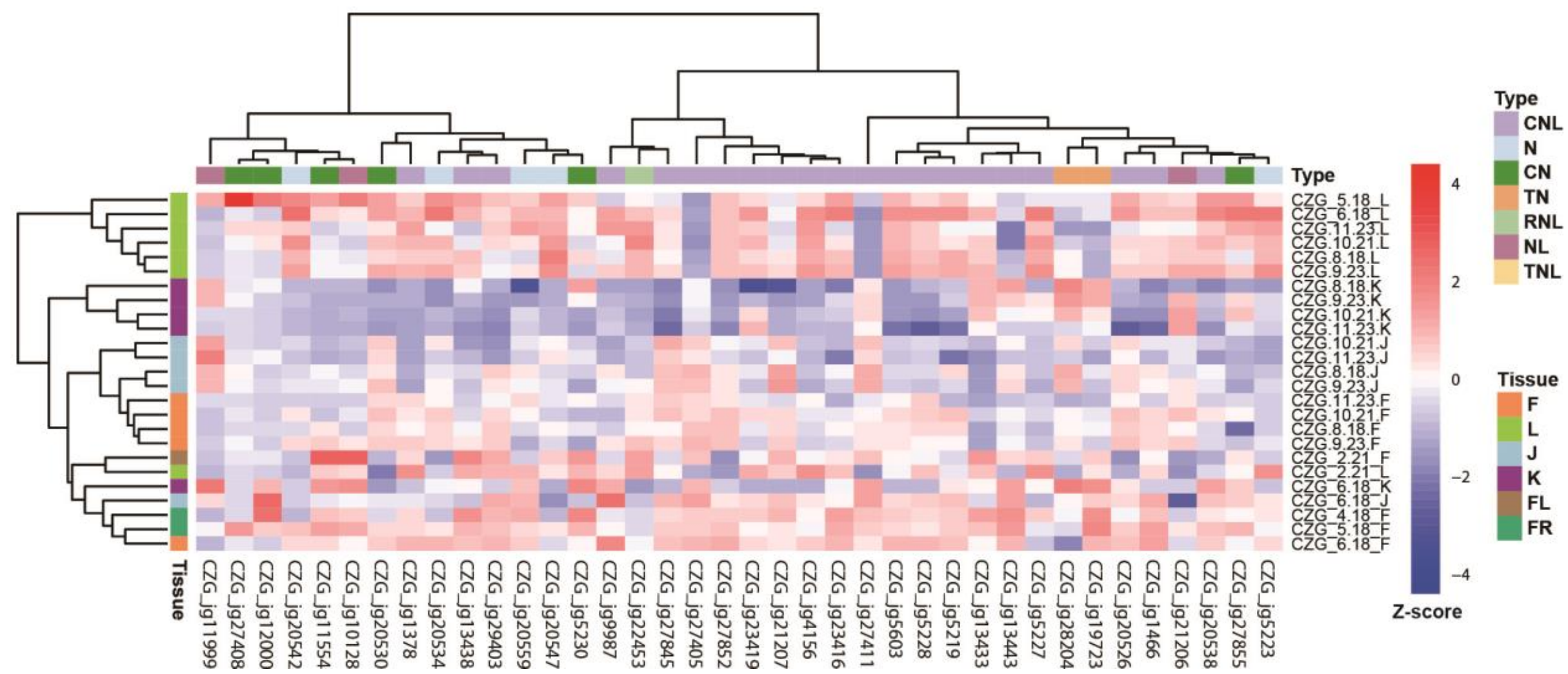

Figure S7. The core NLR gene expression profile in *C. reticulata* cv. Chachiensis. “F”: peel; “L”: leaves; “J”: pulp; “K”: seeds; “FL”: flowers, “FR”: young fruit. Group names in row indicate the sampling time. TNL, TIR-NLR (TNL); CNL, CC-NLR; RNL, CC-RPW8-NLR; N, solo NB-ARC domain; TN, TIR domain combined with NB-ARC domain, and NL, NB-ARC domain combined with leucine-rich repeat.

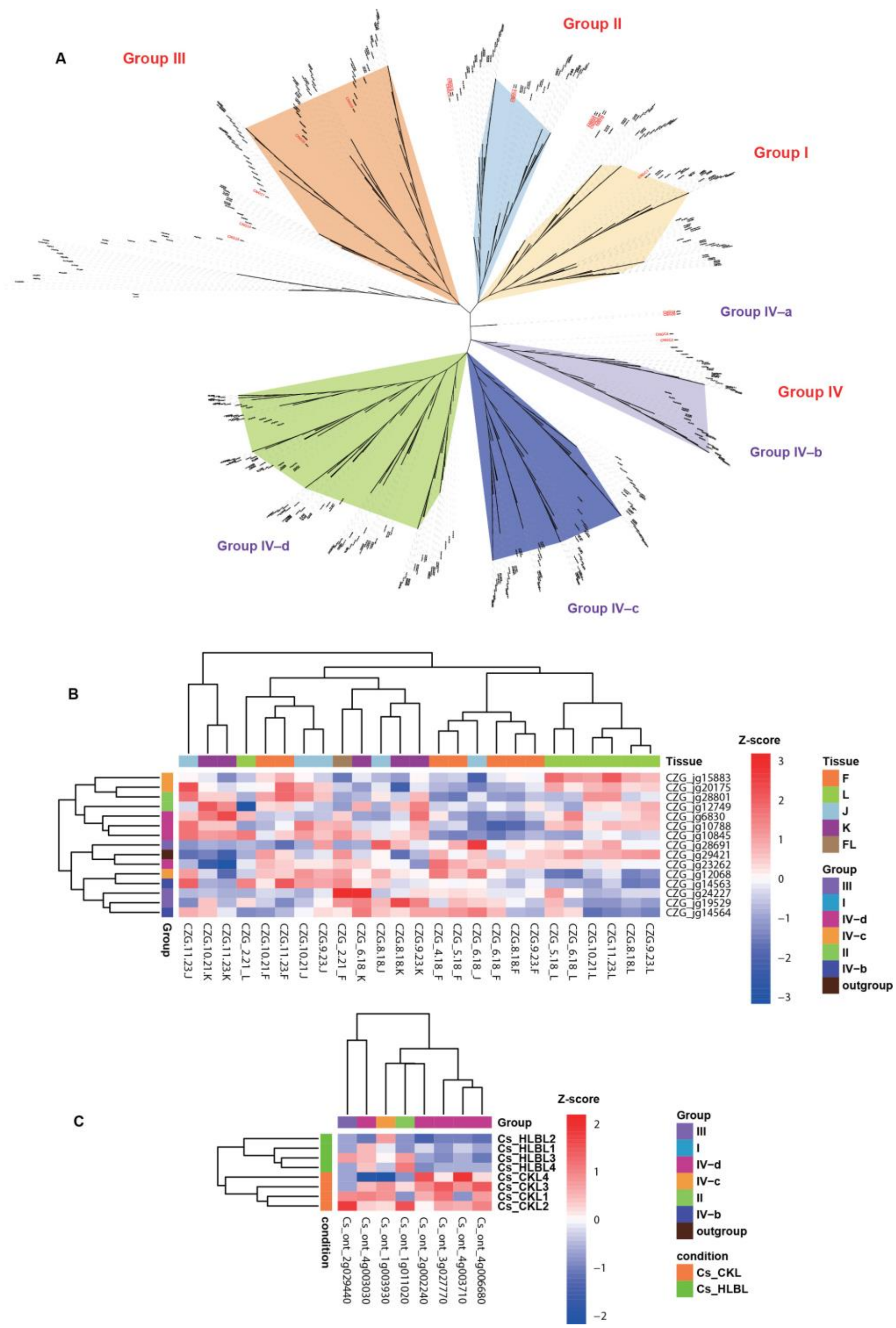

Figure S8. CNGCs in citrus. A, Phylogenetic tree of citrus species and relative's CNGCs and Arabidopsis CNGCs. **B,** The core CNGCs expression profile in *C. reticulata* cv. Chachiensis. "F":young fruit of peel; "L":leaves; "J": pulp; "K": seeds; "FL": flowers. **C,** The expression of CNGC genes in *C. sinensis* leaves in response to CLas infection at 26 week post inoculation. Group names indicate the clades of phylogenetic trees the genes belonged to.

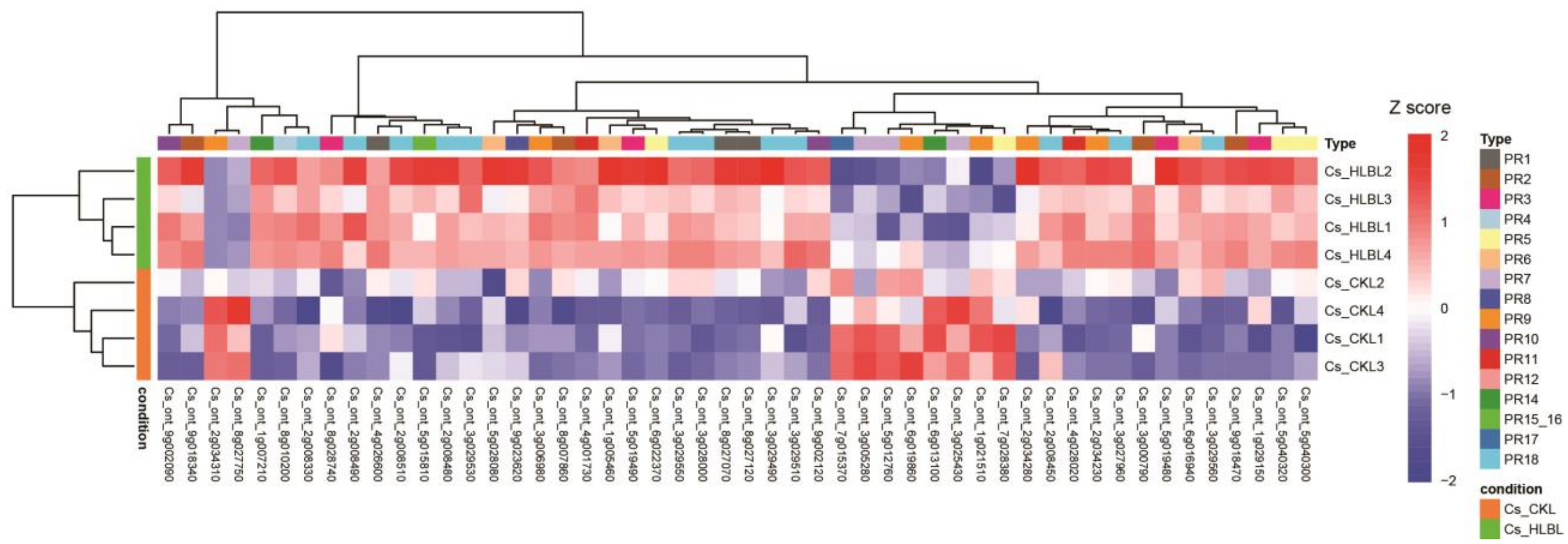

Figure S9. Expression patterns of *PR* genes in *C. sinensis* at 26 weeks post-graft inoculation with CLas. Heatmap showing the scaled expression patterns of *PR* genes in *C. sinensis* leaf under control and HLB conditions at 26 weeks post-graft inoculation with CLas. Columns represent PR genes, with PR family classification indicated by the top annotation bar. Rows represent biological samples, with conditions indicated by the left annotation bar. HLBL: CLas positive leaf. CKL: healthy leaf.

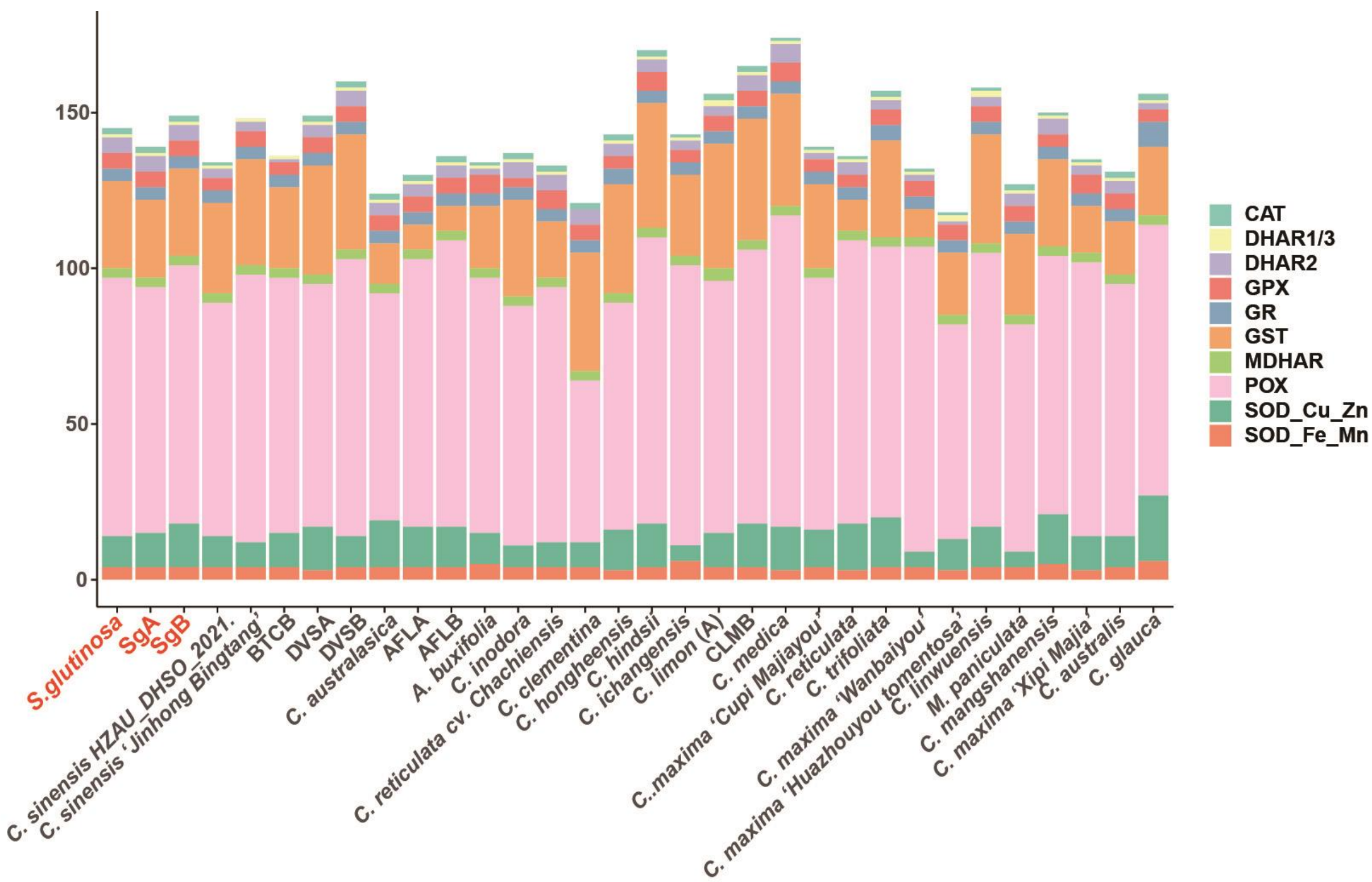

Figure S10. Antioxidant enzymes in citrus. CAT, catalase; DHAR, dehydroascorbic acid reductase; GPX, glutathione peroxidase; GR, glutathione reductase; GST, glutathione S-transferase; MDHAR, monodehydroascorbate reductase; POX, peroxidase; SOD_Cu_Zn, copper_zinc superoxide dismutase; SOD_Fe_Mn, iron_manganese superoxide dismutase

A

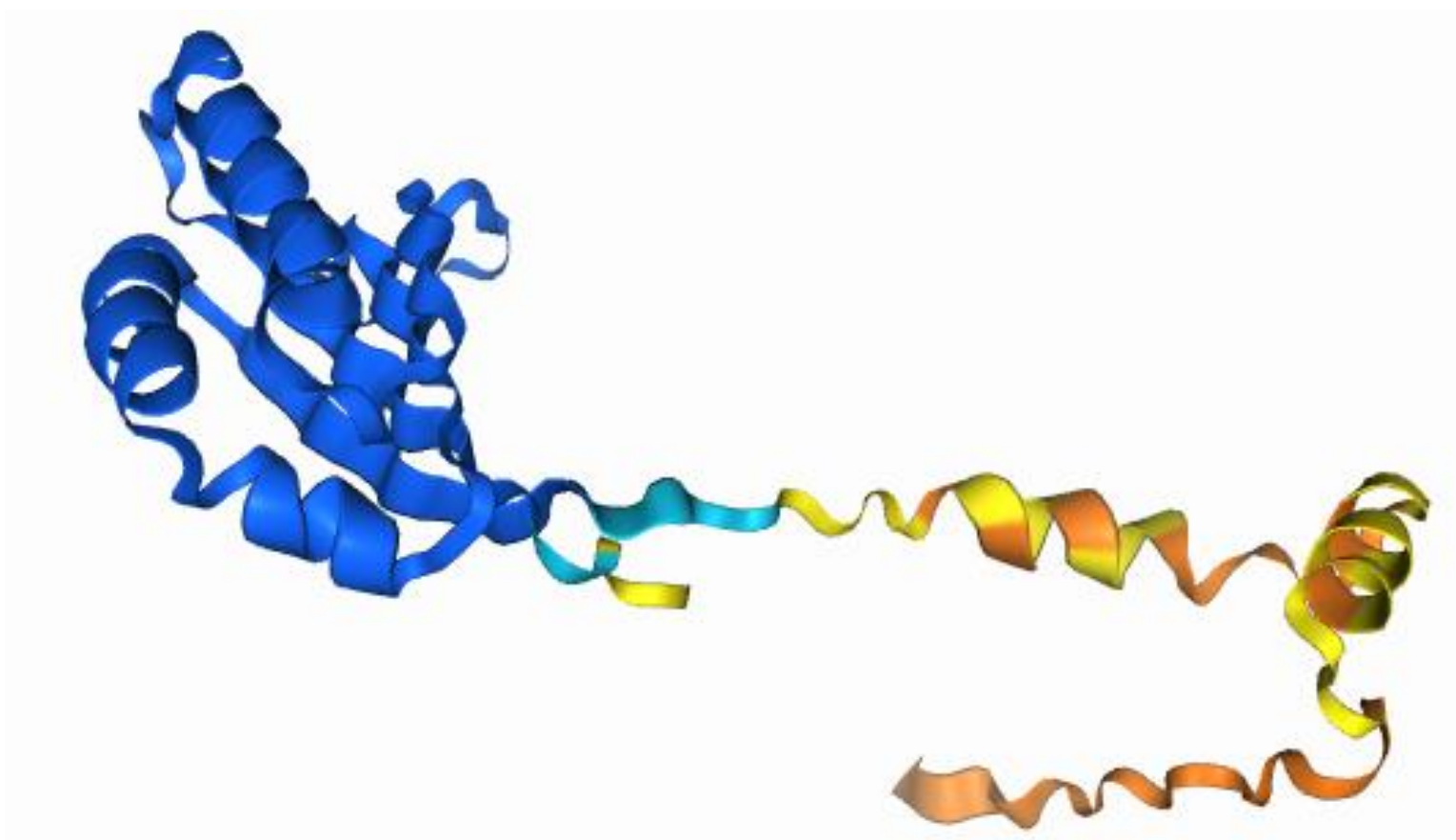

B

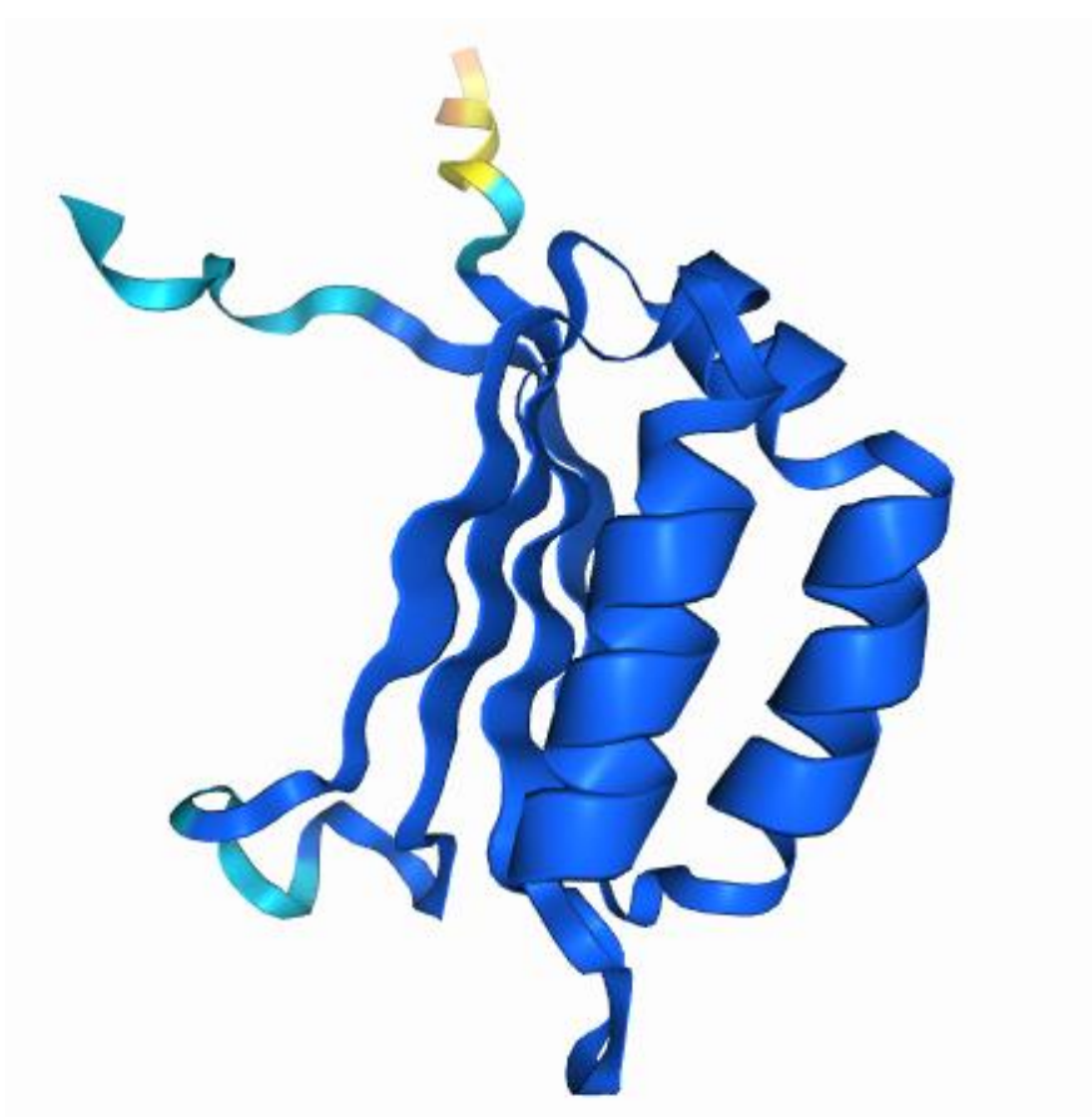

Figure S11. The structure models of the two SAMP homolog proteins. A, The 157-aa homolog protein SWI_jg18875 with the predicted structure score pTM = 0.65. **B,** The 112-aa homolog protein SWI_jg26404 with pTM = 0.84.

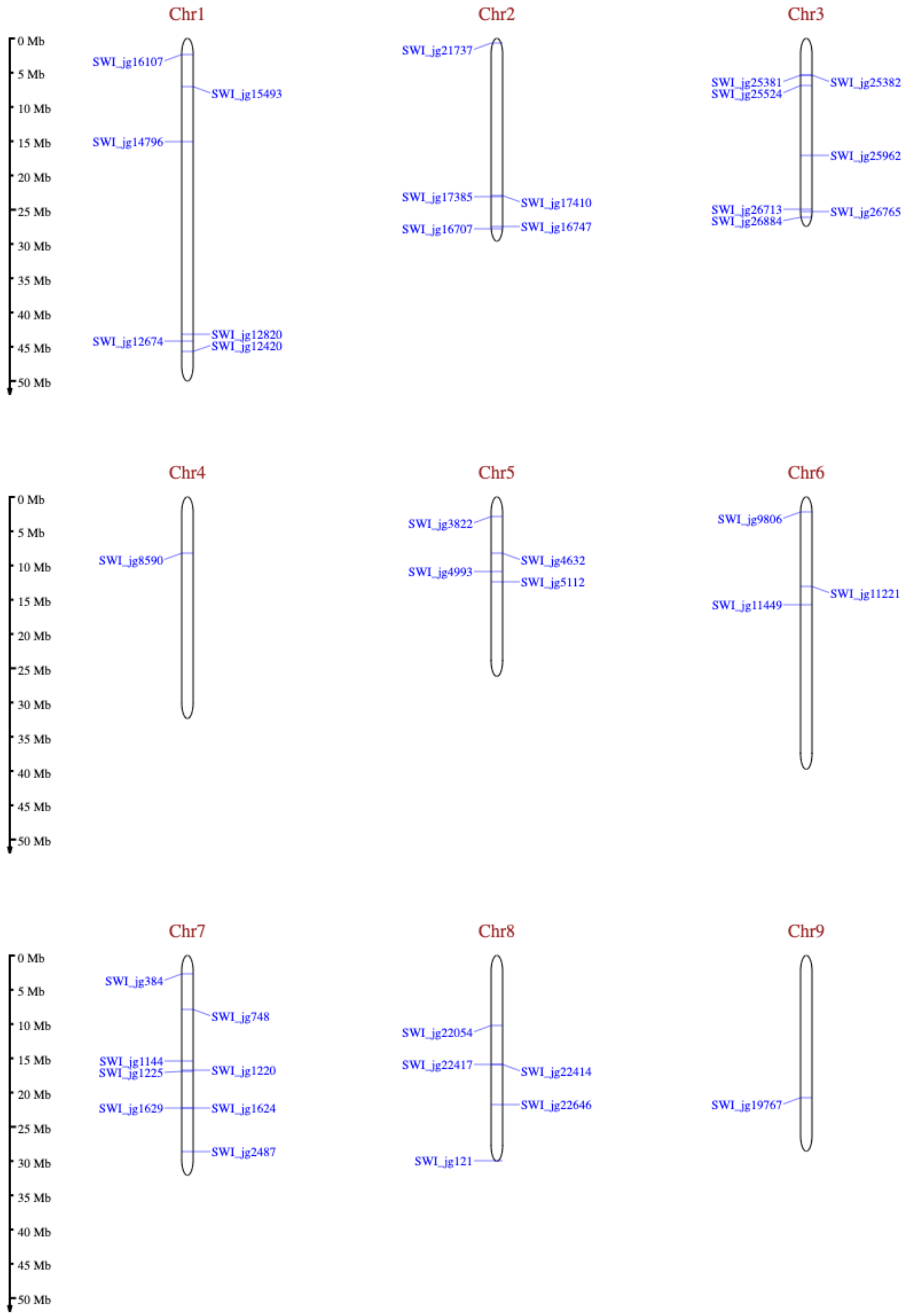

Figure S12. The distribution of AMPs in *S. glutinosa*.

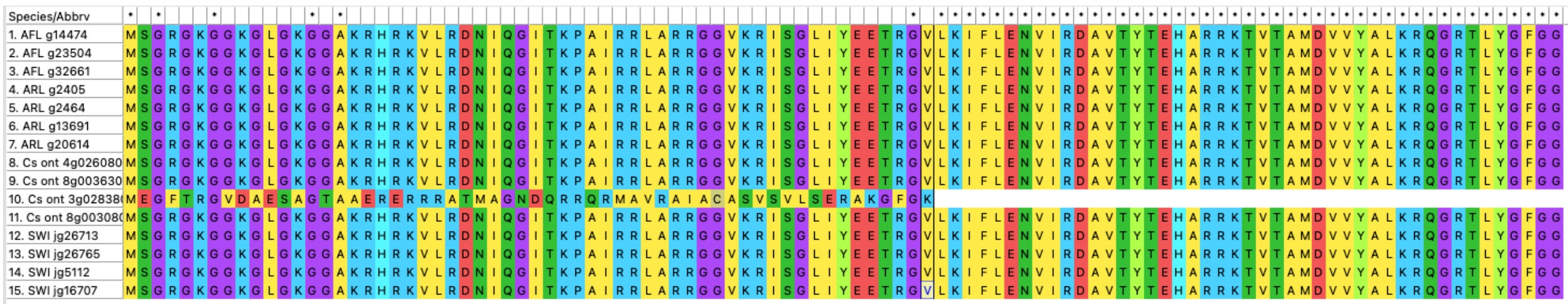

Figure S13. Multiple sequence alignment analysis of the histone H4 derived AMPs.
